# Inflammation in the COVID-19 airway is due to inhibition of CFTR signaling by the SARS-CoV-2 spike protein

**DOI:** 10.1038/s41598-024-66473-4

**Published:** 2024-07-23

**Authors:** Hung Caohuy, Ofer Eidelman, Tinghua Chen, Ognoon Mungunsukh, Qingfeng Yang, Nathan I. Walton, Bette S. Pollard, Sara Khanal, Shannon Hentschel, Catalina Florez, Andrew S. Herbert, Harvey B. Pollard

**Affiliations:** 1 Department of Anatomy, Physiology and Genetics, Uniformed Services University School of Medicine, Uniformed Services University of the Health Sciences, Bethesda, MD 20814 USA; 2 Collaborative Health Initiative Research Program (CHIRP), Uniformed Services University of the Health Sciences, Bethesda, MD 20814 USA; 3 Consortium for Health and Military Performance (CHAMP), Uniformed Services University of the Health Sciences, Bethesda, MD 20814 USA; 4 Center for Military Precision Health, Uniformed Services University of the Health Sciences, Bethesda, MD 20814 USA; 5 Center for the Study of Traumatic Stress (CSTS), and Department of Psychiatry, Uniformed Services University of the Health Sciences, Bethesda, MD 20814 USA; 6 Silver Pharmaceuticals, Rockville, MD 20854 USA; 7 Virology Division, United States Army Medical Research Institute of Infectious Diseases (USAMRIID), Fort Detrick, Frederick, MD 21702 USA; 8 The Geneva Foundation, Tacoma, WA 98402 USA; 9 Cherokee Nation Assurance, Catoosa, OK 74015 USA

**Keywords:** Respiration, Respiratory tract diseases

## Abstract

SARS-CoV-2-contributes to sickness and death in COVID-19 patients partly by inducing a hyper-proinflammatory immune response in the host airway. This hyper-proinflammatory state involves activation of signaling by NFκB, and unexpectedly, ENaC, the epithelial sodium channel. Post-infection inflammation may also contribute to "Long COVID"/PASC. Enhanced signaling by NFκB and ENaC also marks the airway of patients suffering from cystic fibrosis, a life-limiting proinflammatory genetic disease due to inactivating mutations in the CFTR gene. We therefore hypothesized that inflammation in the COVID-19 airway might similarly be due to inhibition of CFTR signaling by SARS-CoV-2 spike protein, and therefore activation of both NFκB and ENaC signaling. We used western blot and electrophysiological techniques, and an organoid model of normal airway epithelia, differentiated on an air–liquid-interface (ALI). We found that CFTR protein expression and CFTR cAMP-activated chloride channel activity were lost when the model epithelium was exposed to SARS-CoV-2 spike proteins. As hypothesized, the absence of CFTR led to activation of both TNFα/NFκB signaling and α and γ ENaC. We had previously shown that the cardiac glycoside drugs digoxin, digitoxin and ouabain blocked interaction of spike protein and ACE2. Consistently, addition of 30 nM concentrations of the cardiac glycoside drugs, prevented loss of both CFTR protein and CFTR channel activity. ACE2 and CFTR were found to co-immunoprecipitate in both basal cells and differentiated epithelia. Thus spike-dependent CFTR loss might involve ACE2 as a bridge between Spike and CFTR. In addition, spike exposure to the epithelia resulted in failure of endosomal recycling to return CFTR to the plasma membrane. Thus, failure of CFTR recovery from endosomal recycling might be a mechanism for spike-dependent loss of CFTR. Finally, we found that authentic SARS-CoV-2 virus infection induced loss of CFTR protein, which was rescued by the cardiac glycoside drugs digitoxin and ouabain. Based on experiments with this organoid model of small airway epithelia, and comparisons with 16HBE14o- and other cell types expressing normal CFTR, we predict that inflammation in the COVID-19 airway may be mediated by inhibition of CFTR signaling by the SARS-CoV-2 spike protein, thus inducing a cystic fibrosis-like clinical phenotype. To our knowledge this is the first time COVID-19 airway inflammation has been experimentally traced in normal subjects to a contribution from SARS-CoV-2 spike-dependent inhibition of CFTR signaling.

## Introduction

SARS-CoV-2 contributes to sickness and death in COVID-19 patients partly by inducing a hyper-proinflammatory state in the host airway^1,2^. Consistently, COVID-19 patients who have been admitted to the Intensive Care Unit (ICU) are found to also have the most severe forms of inflammatory disease^3^. In the COVID-19 lung, this hyper-proinflammatory state, also termed cytokine storm^4^ or cytokine release syndrome^5^, involves both activation of the TNFα/NFκB signaling pathway^6,7^, and the activation of ENaC, the Epithelial Sodium Channel^8,9^. NFκB activation drives increased expression of IL-8, IL-6 and other cytokines and chemokines by epithelial cells lining the host airway^10^. In the COVID-19 lung, NFκB activation thus attracts high levels of neutrophils^11^ and induces pathogenic changes in immune cells^11,12^. Simultaneously, activation of ENaC contributes to inflammation by dehydrating the airway surface and by impairing mucociliary clearance^9,13,14^. However, the mechanisms responsible for the coincident activation of TNFα/NFκB and ENaC signaling, and the ensuing excessive inflammation, are unknown. In addition, although it is widely believed that sustained post-infection inflammation may contribute to Post-Acute Sequelae of COVID-19 (PASC), or “long COVID”, the mechanisms responsible for PASC are also unknown^15–17^. It has been suggested that the innate immune system could contribute to these proinflammatory mechanisms. For example, following viral infection, Pattern Recognition Receptors (PRRs) such as RIG-1-like receptors (RLRs), and endosomal and extracellular Toll-like Receptors (TLRs) can detect single and double stranded RNAs, thereby activating NFκB signaling^18^. In addition, DNA can activate interferon and NFκB signaling by the cGas-STING pathway^19–21^. However, SARS-CoV-2 and other coronaviruses have evolved elaborate escape mechanisms for most known PPR signaling processes^18,20–28^. In addition, while these viral escape mechanisms may be breached in the most severe forms of COVID-19^22^, the coincident activation of ENaC signaling does not appear to be a property of any of these pathways. Finally, it is also possible that the COVID-19 hyper-proinflammatory state could be induced by the binding of viral spike (S) protein to the viral receptor angiotensin converting enzyme 2 (ACE2) on the cell surface^29^. Thus, drugs that block the spike:ACE2 binding reaction might also have therapeutic value. For example, approved cardiac glycoside drugs such as digitoxin, digoxin, and ouabain have been shown to be competitive inhibitors of spike:ACE2 binding^29^. In vitro, these drugs block both cell entry by spike-pseudotyped virus and infection by authentic SARS-CoV-2 virus^29,30^. However, the coincident involvement of TNFα/NFκB and ENaC signaling is not intrinsic to any of these candidate mechanisms, and it may be that the path to understanding the true origins of the pro-inflammatory state in the COVID-19 lung may lie elsewhere.

One possibility for the origin of the COVID-19 pro-inflammatory state is Cystic Fibrosis (CF), a rare, lethal, pro-inflammatory genetic disease, which is characterized by coincident and sustained activation of proinflammatory TNFα/NFκB and ENaC signaling^9,31,32^. Importantly, the proinflammatory activation mechanisms for NFκB and ENaC signaling in CF are well known, and depend on the presence of inactivating mutations in *CFTR*, the Cystic Fibrosis Transmembrane Conductance Regulator^9^. These inactivating mutations in CFTR prevent CFTR from binding and inactivating TRADD (Tumor necrosis factor receptor type 1-associated DEATH domain), the first intracellular adaptor for the apical TNFα/TNFR1complex^33^. Once free from constitutive CFTR inhibition, the now active TRADD signals to IKKα,β to phosphorylate IκBα^33–35^. Upon phosphorylation, IκBα releases hitherto quiescent NFκB, p65 to enter the nucleus where it drives cytokine and chemokine production^33^. Coincidentally, the mutant CFTR is unable to also execute its cAMP-activated chloride channel function, which leads to the activation of the ENaC channel^31,32^. This is because under normal conditions, airway hydration depends on movement Cl^-^ into the airway by CFTR, and coincident movement of Na^+^ counterions by “inactive” ENaC. Normally, water then passively enters the airway by following the osmotic gradient of NaCl^32,36,37^. However, without initial Cl^-^ from mutant CFTR in the airway, the ENaC channel becomes proteolytically activated and removes Na^+^ out from the cell through the basolateral surface^9^. This leaves the airway surface to be injured by dehydration, thus causing further NFκB-dependent inflammation^14,38^. Thus, it might be reasonable to consider whether loss of CFTR function could contribute in some way to COVID-19. However, when a lung epithelial cell loses total CFTR expression, either by CF mutations, or experimentally by chemical or molecular means, the result has been reported to be loss of ACE2 expression and subsequent impairment of SARS-CoV-2 entry ^39^. Proinflammatory signaling due to the missing CFTR can still be observed, but virus-dependent increases are limited. Because CF is so infrequent, the vast majority of people who encounter the SARS-CoV-2 virus would be expressing normal levels of ACE2 and CFTR. Therefore, to elucidate how the SARS-CoV-2 infection affects the CFTR-dependent pro-inflammatory response, our approach was incubate SARS-CoV-2 or viral spike protein with otherwise normal lung epithelial cells, and to test for loss of CFTR and consequent activation of proinflammatory TNFα/NFκB signaling^40^*.*

Further encouraged by the parallel between COVID-19 and CF in terms of activation of both ENaC and TNFα/NFκB signaling, we have hypothesized that inflammation in the COVID-19 airway might be due to inhibition of CFTR signaling in normal lung epithelial cells by the SARS-CoV-2 spike protein. To test this hypothesis, we employed an organoid-based lung-on-a chip platform by differentiating hTERT-transformed BCi.NS1.1 basal stem cells into a model epithelium at the air–liquid-interface (ALI)^41–43^. Attractive features of this model epithelium included (i) histological and molecular fidelity to normal small airway epithelial cell physiology^41,43^; (ii) a normal male karyotype, 46 X/Y^41–43^; (iii) cell specific expression of COVID-19-related genes including ACE2, ADAM10 and ADAM17, TMPRSS2, FURIN and CTSL^43^; and (iv) our preliminary data demonstrating functional expression of CFTR chloride channels and CFTR protein in differentiated epithelia^40^. We then tested the CFTR inhibition hypothesis in differentiated epithelia by investigating whether spike proteins were able to reduce CFTR expression. Next, we tested whether spike-dependent CFTR reduction led to activation of proinflammatory NFκB and ENaC signaling. Finally, we tested whether cardiac glycoside drugs such as digitoxin, digoxin and ouabain could rescue CFTR loss that was due to either spike proteins or authentic SARS-CoV-2. Based on supporting results, we suggest that inflammation in the COVID-19 airway may be initiated by inhibition of CFTR signaling by SARS-CoV-2 spike protein, thus inducing a cystic fibrosis-like proinflammatory clinical phenotype. It is possible that this insight may lead to new approaches to prevention and therapy for COVID-19.

## Results

### SARS-CoV-2 spike protein activates TNFα/NFκB signaling in airway epithelia

**Figure 1:**
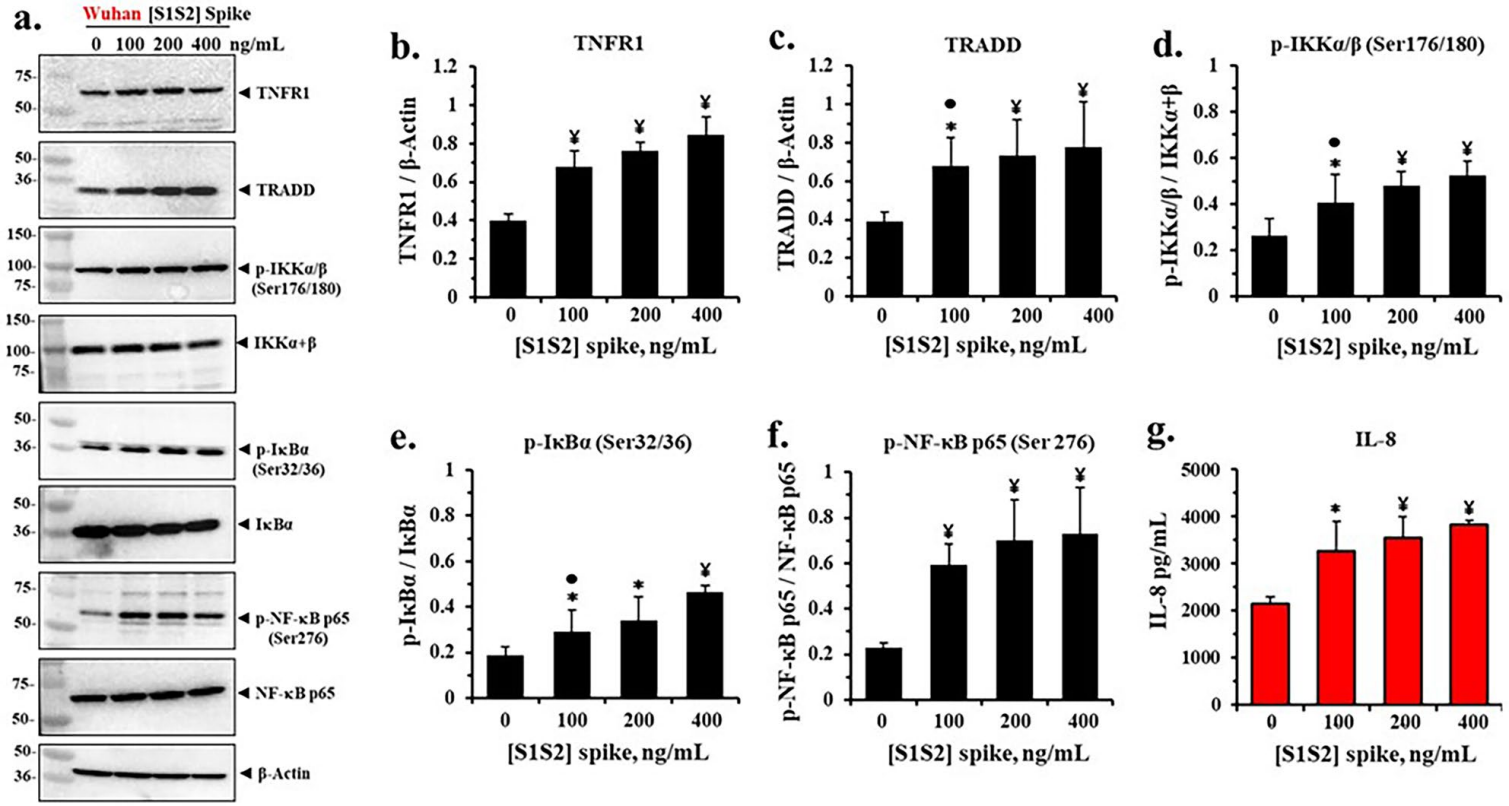
SARS-CoV-2 [S1S2] spike protein activates pro-Inflammatory TNFα/NF-κB signaling proteins and IL-8 expression in differentiated BCi.NS1.1 (d-BCi) epithelia. The apical surfaces of the ALI differentiated dBCi epithelia were treated with different concentrations of Wuhan-Hu-1 [S1S2] spike protein for 4 h, washed and then incubated for an additional 20 h at the Air–Liquid-Interface (ALI) condition. (**a**) Representative western blotting images showing the expression levels of TNFR1, TRADD, phospho-IKKα/β (Ser 176/180), IKKα + β, phospho-IκBα (Ser 32/36), IκBα, phospho-NF-κB p65 (Ser 176), NF-κB p65, and β-Actin. β-actin was used for equal loading of protein. (**b**–**f**) Quantification by densitometric analysis for the data in part (**a**) using BioRad Image Lab software. The data are expressed as mean ± SD (N = 5). (g) IL-8 expression in the subphase of the ALI cultures. Data are presented as means ± SD (N = 3). Statistical *p* values were determined with a one-way ANOVA, followed by both Holm’s and Dunnett’s post-hoc tests comparing each mean to the medium control. For the Holm test, *, *p* < 0.05; and ¥, *p* < 0.01. Dunnett’s tests were consistently significant (*p* < 0.05), except as noted (•).

To test if incubation of differentiated epithelia with spike protein reduced CFTR expression and activated NFκB and its downstream targets, we incubated cells grown in an ALI with different concentrations of the original Wuhan-Hu-1 [S1S2] spike protein. Thereafter, we measured expression levels of a comprehensive set of proteins in the TNFα/NFκB signaling pathway, which we expected to be upregulated. The representative western blots in Fig. 1a showed that as the concentration of spike protein was increased, the protein expressions also increased for TNFR1, TRADD, p-IKKα,β, p-IκBα, p-NFκB p65 and the chemokine IL-8. By contrast, the non-phosphorylated substrate proteins IKKα,β, IκBα, and NFκB p65 were not significantly changed. Figure 1b–f showed that the increases in TNFR1, TRADD, p-IKKα,β, p-IκBα, and p-NFκB p65 were statistically significant. Figure 1g also showed that IL-8, a direct target of p-NFκB p65, was also significantly increased in the liquid sub-phase, in direct proportion to increases in spike protein concentrations applied to the epithelia. Thus, exposure to spike protein dose-dependently activated the proinflammatory steps in the TNFα/NFκB signaling pathway. Since the elevation of TRADD expression in this process is specifically predicted to occur as a consequence of loss of native CFTR from the system^33^, it was therefore possible that loss of CFTR function could be involved in the spike-dependent activation of proinflammatory TNFα/NFκB signaling in airway epithelia.

### Spike protein reduces CFTR chloride channel activity in differentiated airway epithelia

**Figure 2:**
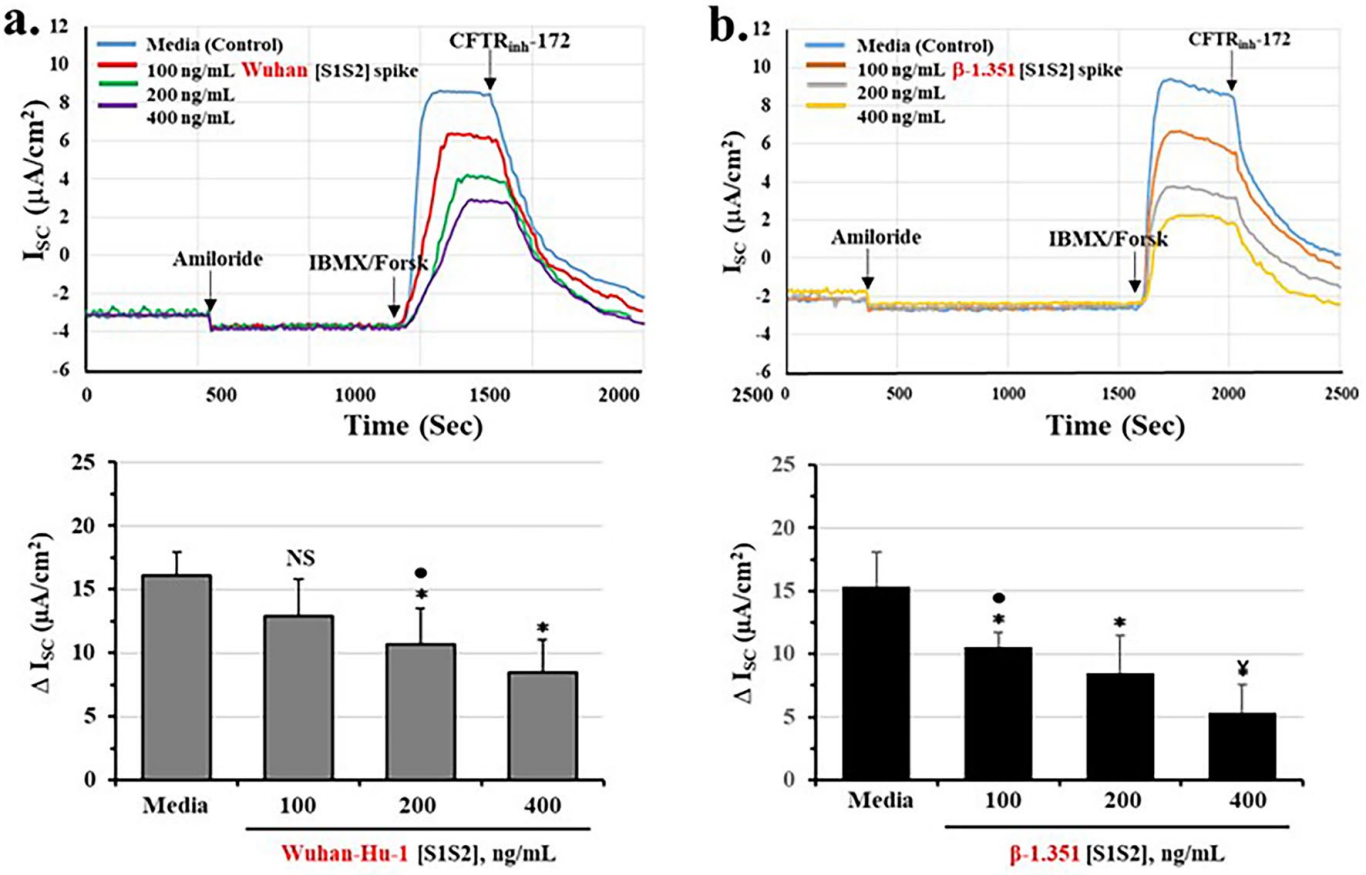
Wuhan-Hu-1 and β-1.351 strain spike proteins dose-dependently reduce cAMP-activated CFTR chloride channel activity in differentiated BCi.NS1.1 (d-BCi) epithelia. (**a**) ALI differentiated dBCi cells were incubated apically with different concentrations of Wuhan-Hu-1 [S1S2] spike protein for 4 h, washed and then incubated for additional 20 h under ALI conditions. CFTR-dependent short-circuit currents (*I*_sc_) were measured in Ussing Chambers as the changes in response to Amiloride, IBMX/Forskolin, and CFTR_inh_-172. Representative *I*_sc_ tracings (*panel top*) and summary of changes in *I*_sc_ of four independent Ussing chamber experiments (*bottom panel*) are shown. (**b**) dBCi cells were incubated with different concentrations of β-1.351 [S1S2] spike protein and CFTR-dependent *I*_sc_ was analyzed as in part (**a**). The data are expressed as means ± SD (N = 4). Statistical *p* values were determined with a one-way ANOVA, followed by both Holm’s and Dunnett’s post-hoc tests comparing each mean to the medium control for the Holm test, *, *p* < 0.05; and ¥, *p* < 0.01. Dunnett’s tests were consistently significant (*p* < 0.05), except as noted (•).

To test whether spike proteins affected CFTR channel function, we analyzed spike-treated epithelia using the Ussing Chamber method. Figure 2a showed that under control conditions, cAMP-activated CFTR chloride channels could be detected, and could be specifically blocked by the CFTR channel inhibitor CFTR_inh_-172. Consistently, we found that when epithelial cultures were incubated with Wuhan-Hu-1 [S1S2] spike protein, CFTR chloride channel activity was significantly and dose-dependently reduced. The inhibition constant (Ki) was calculated to be 446 ng/ml (see Supplemental Fig. S2 and Table 1). To test for the specificity of the spike effect, we next replaced the Wuhan-Hu-1 [S1S2] spike protein with the more potent β-1.351 [S1S2] spike protein^29^. As shown in Fig. 2b, the β-1.351 [S1S2] spike also reduced CFTR channel activity. However, the inhibition constant, K_i_, was 210 ng/ml, or 47% lower than for the Wuhan-1-Hu [S1S2] spike protein (see Supplemental Fig. S2 and Table 1). To further test for the specificity of the spike effect, we treated epithelia with Wuhan-Hu-1 [S1S2] spike protein in the presence of an anti-spike antibody. As shown in Supplemental Fig. S3a–c, the anti-spike antibody completely neutralized the Spike inhibitory effect on both CFTR channel activity and CFTR protein expression. These data thus directly support the concept that spike protein reduces CFTR function.

**Table 1:** Inhibition constants for Wuhan-Hu-1 and β-1.351 [S1S2] spike effects on CFTR channels and CFTR protein, and on binding constants for ACE2.

Spike variant	K_i_, CFTR channel (R^2^) spike, ng /ml	K_i_, CFTR protein (R^2^) spike, ng /ml
Wuhan-Hu-1, [S1S2]	446 (0.9898)	399 (0.9842)
β-1.351 [S1S2]	210 ((0.9590)	193 (0.9590)
Ratio, % Wuhan-Hu-1/β-1.351	(47%)	(48%)

### Spike protein reduces CFTR protein expression in differentiated airway epithelia

**Figure 3:**
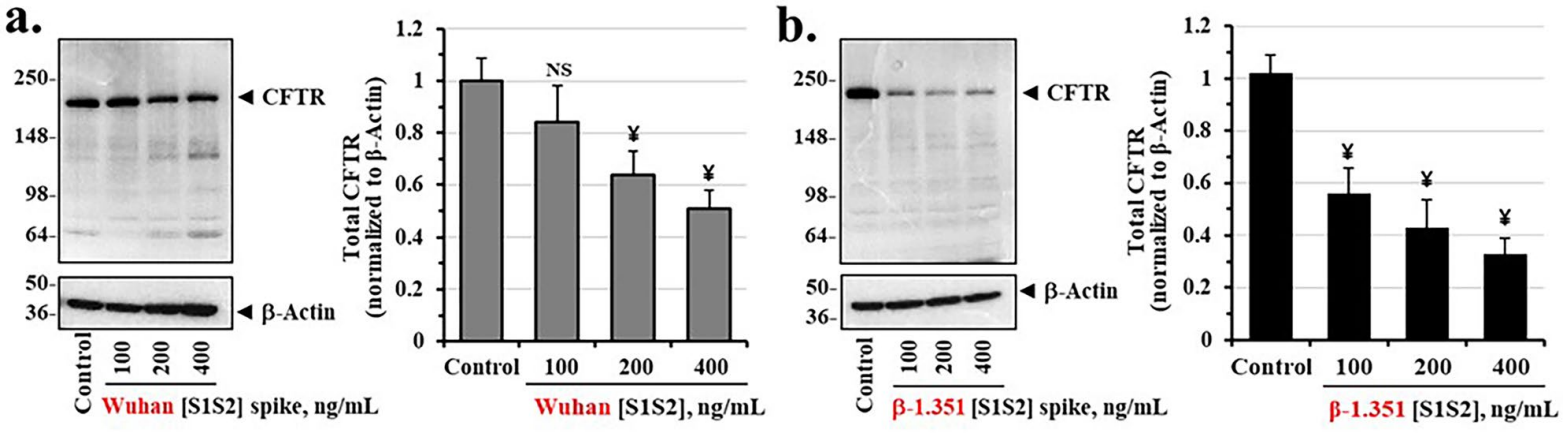
Wuhan-Hu-1 [S1S2] spike and β-1.351 [S1S2] spike proteins suppress CFTR protein expression in differentiated BCi.NS1.1 (d-BCi) epithelia. (**a**) ALI differentiated dBCi cells were incubated apically with different concentrations of Wuhan-Hu-1 [S1S2] spike protein for 4 h, washed and then incubated for additional 20 h under ALI conditions. Representative Western blot images (*left panel*) and densitometric analysis of the expression levels of CFTR (*right panel*) in treated dBCi cells after Ussing chamber analyses are shown. β-actin was used for equal loading of protein. (**b**) dBCi cells were incubated with different concentrations of β-1.351 [S1S2] spike protein and analyzed as in part (**a**). The data are expressed as means ± SD (N = 4). Statistical *p* values were determined with a one-way ANOVA, followed by both Holm’s and Dunnett’s post-hoc tests comparing each mean to the medium control. For the Holm test, *, *p* < 0.05; and ¥, *p* < 0.01. Dunnett’s tests were consistently significant (*p* < 0.05), except as noted (•).

To determine if spike-dependent loss of CFTR chloride channel activity might be due to loss of CFTR protein we incubated epithelia with increasing concentrations of Wuhan-Hu-1 [S1S2] spike and β-1.351 [S1S2] spike proteins, and quantified total epithelial CFTR protein. Figure 3a,b showed that both spike proteins reduced total CFTR. However, the β-1.351 [S1S2] spike protein more potently reduced CFTR concentrations. We also noted that the patterns of reduction in concentration of both Wuhan-Hu-1 and β spike proteins were remarkably similar to those for suppression of the cAMP-activated CFTR chloride channel. The K_i_ values for Wuhan-Hu-1 [S1S2] spike and β-1.351 [S1S2] spike proteins, respectively were 399 ng/ml and 193 ng/ml. The K_i_ value for β-1.351 [S1S2] spike protein was thus reduced relative to the original Wuhan-Hu1-1 [S1S2] spike protein by 48%. The measurements of the kinetic values for CFTR channel and protein are shown in Supplemental Fig. S2 and calculations are summarized in Table 1. The spike protein-dependent loss of CFTR channels may therefore be due to primary loss of CFTR protein.

To test for the generality of spike effects on the BCi-NS1.1 epithelia we tested the two different spike protein variants on primary human bronchial epithelial (HBE) cells. As shown in Supplemental Fig. S4, HBE cells were differentiated at the ALI and treated with either Wuhan Hu-1 [S1S2] or β-1.351 [S1S2] spike protein. As first observed with BCi-NS1.1 epithelia in Fig. 3, the β-1.351 [S1S2] spike protein was more potent than the Wuhan-1-Hu [S1S2] spike protein at reducing cAMP-activated CFTR channel activity, and also more potent at reducing total CFTR protein levels (Supplemental Fig. S4). These results show that differentiated hTERT-transformed BCi-NS1.1 epithelia and primary HBE cells respond similarly to different spike proteins by parallel loss of CFTR protein and CFTR chloride channel activity.

### Cardiac glycoside drugs block spike-dependent loss of CFTR channel activity and CFTR protein expression

**Figure 4:**
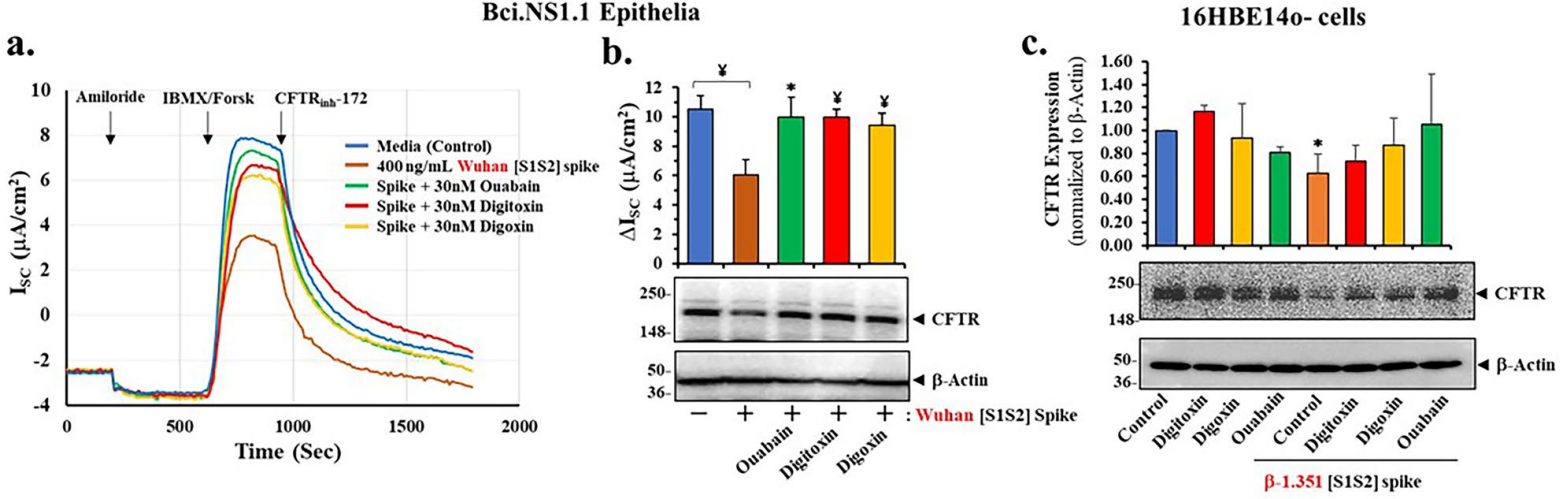
Cardiac glycosides block the inhibitory effect of SARS-CoV-2 [S1S2] spike on cAMP-activated CFTR chloride channels in differentiated BCi.NS1.1 (d-BCi) epithelia and 16HBE14o-cells. (**a**) ALI differentiated dBCi cells were incubated apically with 400 ng/mL Wuhan-Hu-1 [S1S2] spike protein for 4 h in the presence of 30 nM ouabain, digitoxin or digoxin, washed and then incubated for additional 20 h under ALI conditions. CFTR-dependent short-circuit currents (*I*_sc_) were measured in Ussing Chambers as the changes in response to Amiloride, IBMX/ Forskolin, and CFTR_inh_-172. (**a**) Representative current *I*_sc_ tracings and (**b**) summary of changes in *I*_sc_ of four independent Ussing chamber experiments are shown along with a representative image of CFTR Western blot image of treated cells after Ussing chamber analyses (*lower panel*). (**c**) Submerged 16HBE14o- cell cultures were treated with 400 ng/ml β-1.351 [S1S2] spike protein in the presence or absence of 50 nm digitoxin, digoxin or ouabain for 4 h in Serum-free αMEM medium, then washed out with the serum-free αMEM medium, incubated in the complete αMEM medium for additional 20 h. CFTR quantitative analyses normalized with β-Actin values (*top panel*) and a representative CFTR Western blot image (*lower panel*) are shown. β-actin was used for equal loading of protein. The data are expressed as means ± SD (N = 3–4). Statistical *p* values were determined with a one-way ANOVA, followed by both Holm’s and Dunnett’s post-hoc tests comparing each mean to the medium control. For the Holm test, *, *p* < 0.05; and ¥, *p* < 0.01. Dunnett’s tests were consistently significant (*p* < 0.05), except as noted (•).

Cardiac glycosides such as ouabain, digitoxin and digoxin have been shown to be potent competitive inhibitors of ACE2 binding to the SARS-CoV-2 spike protein^29^. The proportional changes in CFTR channel inhibition constants for Wuhan-Hu-1 [S1S2] spike and β-1.351 [S1S2] spike proteins suggest that CFTR channel reduction may be initiated by binding of spike protein to ACE2. We therefore predicted that the cardiac glycosides would block spike-induced reduction of CFTR channel activity. As anticipated, coincubation of Wuhan-Hu-1 [S1S2] spike proteins with 30 nM ouabain, digitoxin or digoxin were found to prevent loss of CFTR activity in differentiated epithelial cells (Fig. 4a). As shown in Fig. 4b prevention of channel activity loss by spike protein exposure was significant for all three cardiac glycoside drugs. We also tested whether the cardiac glycoside drugs could rescue spike-dependent loss of CFTR protein expression in human bronchial lung epithelial 16HBE14o- cells. As shown in Fig. 4c, [S1S2] spike protein from the β-1.351 SARS-CoV-2 virus significantly reduced expression of CFTR protein in these cells. In addition, co-incubating spike-treated cells with either of the three cardiac glycosides resulted in significant rescue of CFTR expression. By contrast, these drugs had little effect on control levels of CFTR (Fig. 4c). Thus, based on both kinetic data and the use of cardiac glycosides as a tool to identify spike:ACE2 binding, it is possible that a direct spike interaction with ACE2 might be initially responsible for loss of CFTR channel activity.

### Spike protein reduces transfected CFTR protein expression in non-lung cystic fibrosis cells

To further test for the possibility of spike effects on transfectionally rescued CFTR in a non-lung cell, we tested spike protein effects on [wildtype]CFTR protein expression in pancreatic ductal CFPAC-1-(4.7/pLJ6)^44^. CFPAC-1 cells are homozygous [*F508del*]*CFTR*, which have been subsequently transfected with the pLJ6 retrovirus bearing [*wildtype*]*CFTR*^45^. Preliminary studies had shown that CFPAC-1 (4.7/pLJ6) cells expressed ACE2. Supplemental Fig. S5a, and the summary bar graphs in Supplemental Fig. S5c, showed that [S1S2] spike proteins from the Wuhan-Hu-1 and β-1.351 SARS-CoV-2 significantly reduced expression of recombinant CFTR protein in these cells. In addition, co-incubating spike-treated cells with cardiac glycosides resulted in rescue of CFTR expression by digitoxin, digoxin and ouabain. By contrast, these drugs had little effect on control levels of CFTR, as shown in Supplemental Fig. S5b and summary bar graphs in Supplemental Fig. S5d. These results thus show that spike protein-dependent loss of CFTR, and rescue of lost CFTR by cardiac glycoside drugs, can be detected in epithelial cells other than lung, and in cells for which CFTR is recombinant rather than native.

### CFTR co-immunoprecipitates with ACE2 in basal stem cells and differentiated epithelia

**Figure 5:**
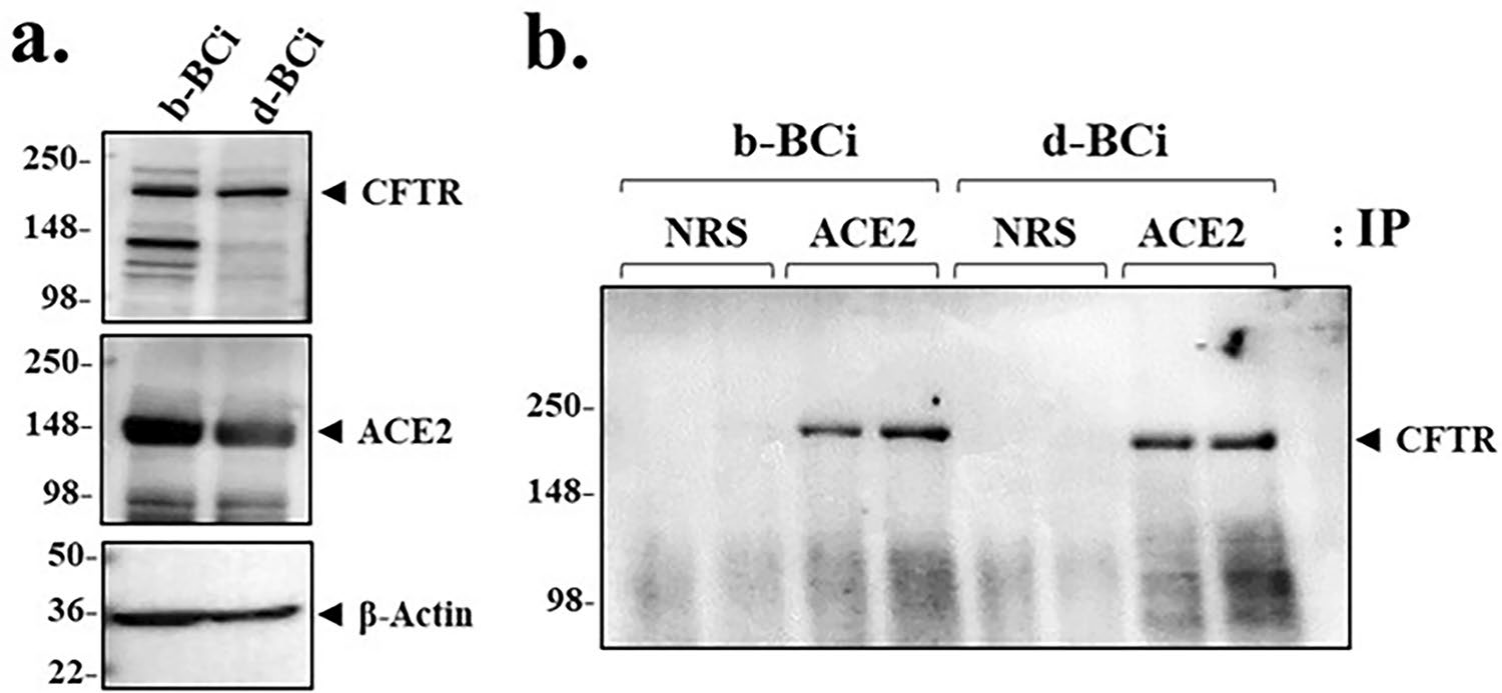
ACE2 and CFTR co-immunoprecipitate in both basal cells and differentiated BCi.NS1.1 (d-BCi) epithelia. (**a**) A representative western blot image shows both untreated basal (b-BCi) cells and differentiated (d-BCi) cells expressing CFTR and ACE2. β-actin was used for equal loading of protein. (**b**) A representative western blot image showing CFTR co-immunoprecipitating with ACE2 from both basal (b-BCi) and differentiated (d-BCi) epithelia lysates. Normal rabbit serum (NRS) was used as the control. Western blot data represent the results of three independent experiments.

To further test whether ACE2 physically interacted with CFTR, we tested if CFTR and ACE2 could co-immunoprecipitate. Figure 5a showed that both BCi.NS1.1 basal cells and differentiated BCi.NS1.1 epithelia contain ACE2 and CFTR. In native airways CFTR is present in secretory cells, followed by basal cells, and infrequently by ionocytes^46^. Consistently, Fig. 5b showed that when ACE2 was first immunoprecipitated from either basal or epithelial cell lysates, both immunoprecipitates contained CFTR. Next, to test whether spike protein affected CFTR in the ACE2:CFTR co-immunoprecipitate, we incubated the epithelia with the Wuhan-Hu-1 [S1S2] spike protein, and then measured co-immunoprecipitation of ACE2 and CFTR with anti-ACE2 antibody. The total lysate lanes in Supplemental Fig. S6a showed that increasing concentrations of the Wuhan-Hu-1 [S1S2] spike protein caused a dose-dependent reduction in CFTR, but an increase in ACE2. These results are paralleled by spike-dependent loss of CFTR in co-immunoprecipitated ACE2:CFTR (Supplemental Fig. S6b). Thus it is possible that when [S1S2] spike protein binds ACE2, it causes release of bound CFTR from the ACE2:CFTR complex, leaving CFTR to be destroyed by a subsequent process. Importantly, these data do not exclude the possibility that ACE2 might be a physical bridge, direct or indirect, between spike protein and CFTR.

### Spike protein inhibits CFTR recovery from endosomal recycling

**Figure 6:**
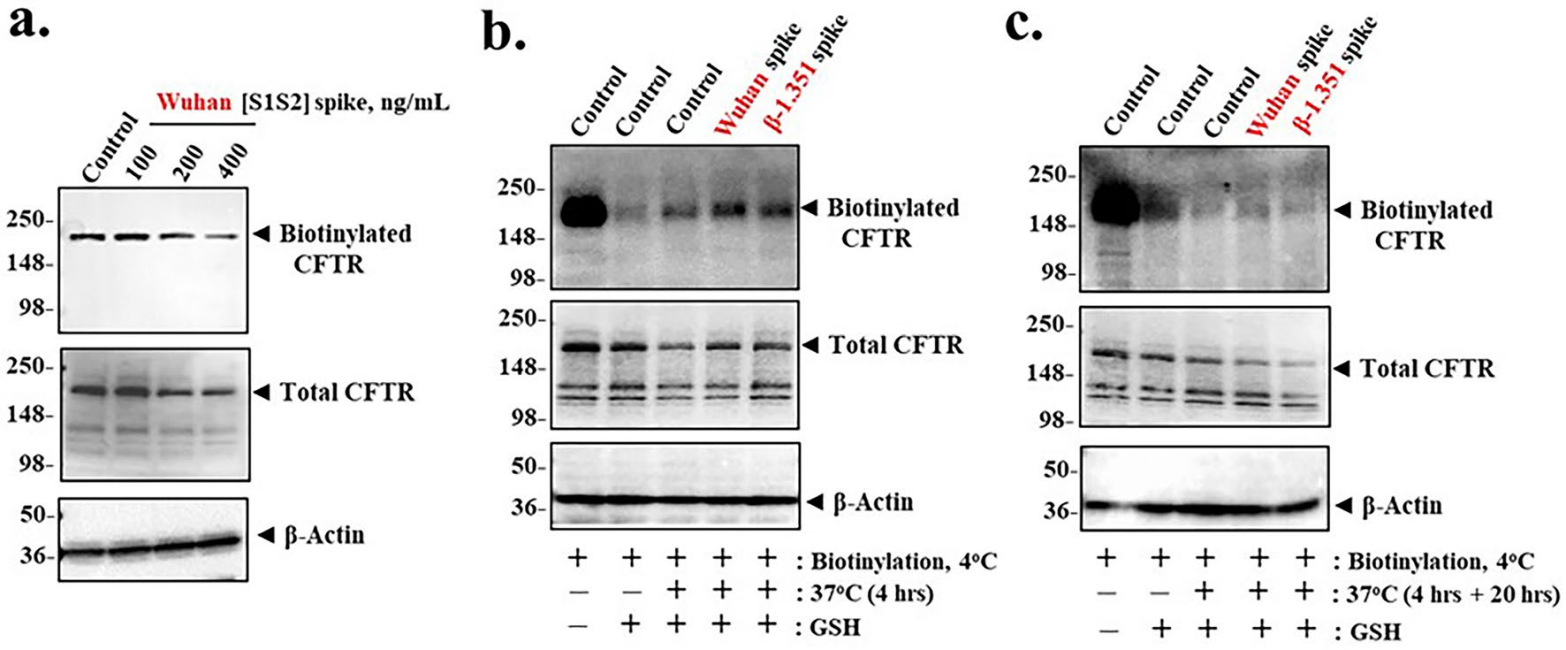
Spike proteins induce loss of cell surface CFTR expression by inhibiting endosomal recycling in differentiated BCi.NS1.1 (d-BCi) epithelia. (**a**) ALI differentiated dBCi cells were incubated apically with different concentrations of Wuhan-Hu-1 [S1S2] spike protein for 4 h, washed and then incubated for additional 20 h under ALI conditions. After completion of incubation, apical membrane expression of CFTR was determined by cell surface biotinylation using cell impermeable Sulfo-NHS-SS-biotin. Representative Western blot images of cell surface and total CFTR expression from three independent experiments are shown. β-actin was used for equal loading of protein (**b**) Recycling assay of endogenous CFTR after 4-h incubation with spike protein. ALI differentiated dBCi cells were first biotinylated at 4 °C with Sulfo-NHS-SS-biotin for 1 h, followed by incubation at 37 °C for 4 h apically with media control or either 400 ng/mL Wuhan-Hu-1 [S1S2] or β-1.351 [S1S2] spike proteins. After incubation, biotin molecules remaining at the cell surface were stripped with glutathione (GSH). (**c**) Recycling assay of endogengous CFTR after a total 24-h incubation. Cell surface biotinylation and treatments were carried out as described in part (**b**), except that after, 4-h incubation with media or spike protein apically, dBCi cells were further incubated at 37 °C for additional 20 h, followed by GSH stripping. Biotinylated and total CFTR pools in parts (**b**) and (**c**) were analyzed by Western blotting. Cell lysate samples (*lane 1*) and samples treated with GSH (*lane 2*) were used as positive controls for biotinylation and GSH stripping processes, respectively. β-actin was used for equal loading of protein. Western analyses represent the results of three independent experiments.

Under normal conditions, CFTR on the plasma membrane is subject to constitutive endosomal recycling in which misfolded or otherwise damaged CFTR is diverted towards destruction in the lysosome^47–49^ (see Supplemental Fig. S7^48^). By this mechanism *ca*. 33% damaged or misfolded CFTR can be lost to the lysosome in 10 min, while the remaining 67% is returned to the apical plasma membrane^47^. To test whether spike-induced loss of apical CFTR was due to failure of recovery from endosomal recycling, we used an impermeant biotin labelling method to ask how much CFTR from the apical surface was returned to the apical surface after exposure to progressively increasing concentrations of spike protein. Figure 6a showed that when apical CFTR was labelled *after* exposure to Wuhan-Hu-1 [S1S2] spike protein, there was a dose-dependent reduction in recovery of recycled CFTR over a 24-h period. This mechanism for native CFTR loss due to exposure to spike protein may thus be consistent with failure of endosomal recycling to return spike protein-treated apical CFTR to the apical plasma membrane.

Consistently, Fig. 6b showed that when apical CFTR was *pre-labeled* with impermeant biotin, and then incubated for 4 h at 37 °C with either Wuhan-Hu-1 or β-1.351 [S1S2] spike protein, there was an increase in glutathione (GSH)-resistant, intracellular biotinylated CFTR. The classical interpretation of this result is that labeled CFTR is immobilized in endosomes, and that the biotin linked to endosomal CFTR by an S–S bond is therefore not available for hydrolysis by GSH. It was therefore possible that when Spike protein binds to the [ACE2:CFTR] complex, CFTR may become marked as "damaged " and thus directed to the lysosome. In further support of this interpretation, we found that after an additional 20-h incubation at 37 °C, the GSH-resistant, biotinylated CFTR that was initially in endosomes was now substantially destroyed (Fig. 6c). Interestingly, there was still a very small biotinylated CFTR signal from the epithelia treated with Wuhan-Hu-1 spike protein, but much less of a signal from epithelia treated with β-1.351 spike protein. By comparison, the 24-h time point for the post-labeled experiment in Fig. 6a, indicated that there was substantially less apical CFTR after the 400 ng/ml spike protein treatment (Fig. 6a). Consistently, the pre-labeled epithelia experiment also indicated that there was substantially less endosomal CFTR left after the 400 ng/ml spike protein treatment (Fig. 6c). Together, these experiments indicate that by the 24 h time point there is substantially less CFTR left in either apical or endosomal compartments after spike protein treatment. Thus, exposure to spike protein inhibits recovery of apical CFTR from the endosomal recycling process.

### Spike protein induces proteolytic activation of ENaC

**Figure 7:**
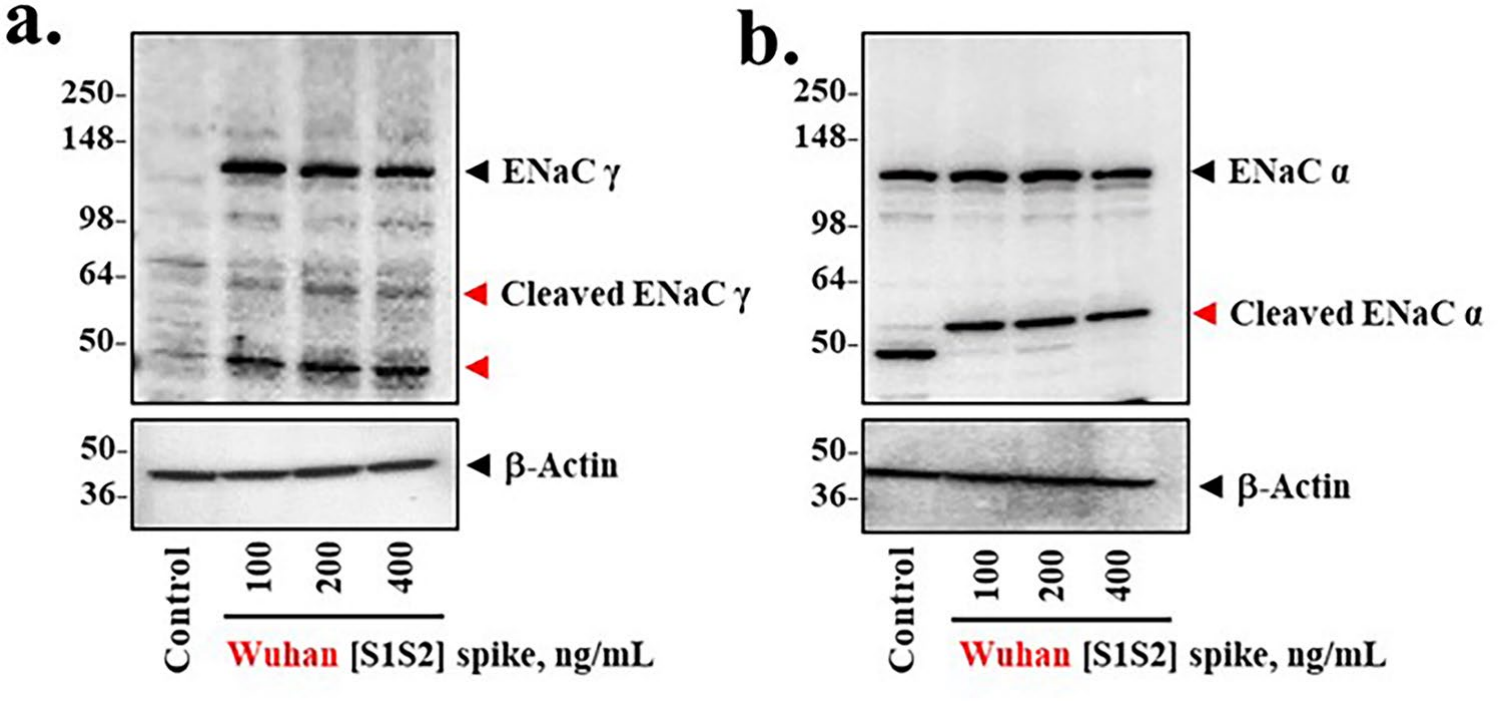
SARS-CoV-2 [S1S2] spike induces proteolytic activation of α and γ ENAC in differentiated BCi-NS1.1 (d-BCi) epithelia. ALI differentiated dBCi cells were incubated apically with different concentrations of Wuhan-Hu-1 [S1S2] spike protein for 4 h, washed and then incubated for additional 20 h under ALI conditions. (**a**) A representative Western blot image showing the detecting levels of total γ ENaC and two proteolytic cleavage products (red arrowheads). (**b**) A representative Western blot image showing the detecting levels of total α ENaC and its proteolytic cleavage product (red arrowhead). β-actin was used for equal loading of protein. Western blots are representative of the results from three independent experiments.

Activation of ENaC occurs when the constitutive inhibitory activity of CFTR is lost, and sodium conductance is activated by proteolytic cleavage of the α and γ chains of the heterotrimeric ENaC channel^9,13,14^. TMPRSS2, FURIN and possibly other serine proteases are responsible^50,51^. To test whether spike protein treatment could activate ENaC, we asked whether proteolytic fragments of activated ENaC subunits could be detected following treatment of differentiated epithelial cells with Wuhan-Hu-1 [S1S2] spike protein. Figure 7a,b showed that over the 20-h period following the 4-h exposure to spike proteins, specific proteolytic fragments were detected for both γ ENaC and α ENaC. Furthermore, additional γ ENaC appears to have been both activated and continuously synthesized. We conclude that spike-dependent loss of CFTR appears to be accompanied by proteolytic activation of both α ENaC and γ ENaC.

### Cardiac glycoside drugs rescue CFTR from loss by authentic SARS-CoV-2 virus

**Figure 8:**
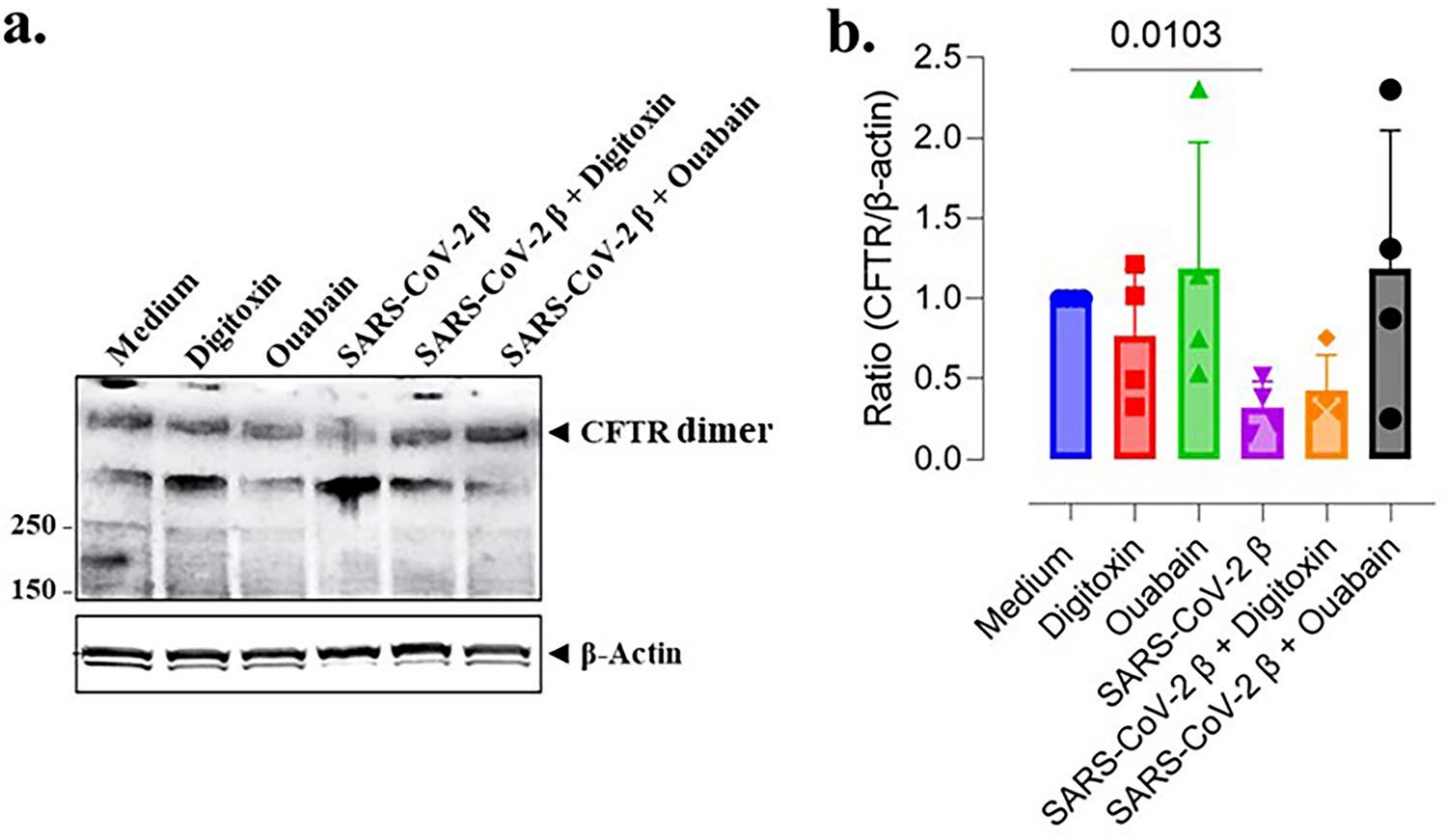
Cardiac glycoside drugs rescue CFTR from loss in differentiated BCi.NS1.1 (d-BCi) epithelia following infection by authentic SARS-CoV-2 virus. (**a**) Representative Western blot of protein samples collected from differentiated dBCi cells, cultured at the ALI for 28 days, and infected with SARS-CoV-2 (strain β 1.315). Conditions included medium only, virus only, and virus with 50 nM of either digitoxin or ouabain. Heating and disulfide bond breakage are omitted from the protein preparation, leaving the high molecular weight CFTR dimer to be detected. β-actin is used as a loading control. (**b**) Densitometry analysis was used to determine CFTR/β-actin ratio. Averages ± SD were calculated from N = 4 independent experiments. *P* values were determined with a one-way ANOVA, followed by a Dunnett’s post-hoc test comparing each mean to the medium control. A significant difference from the medium control was determined only for treatment with spike protein alone (*p* = 0.0103).

We have previously reported that cardiac glycoside drugs such as digitoxin, ouabain and digoxin significantly blocked entry of both Wuhan-Hu-1 spike-pseudotyped virus and of authentic virus into human lung A549 lung epithelial cells^29^. We therefore exposed differentiated lung epithelia to an authentic beta strain of the SARS-CoV-2 virus, in the presence and absence of cardiac glycosides. These first experiments indicated that exposure to the virus was causing too many bands to occur in the CFTR monomer region when analyzed by western blot. We could not therefore be certain about identifying the intact CFTR monomer. However, CFTR exists physiologically as a dimer^52–54^, and the monomer on western blots that we and others detect is a useful artifact caused by detergents, high temperature and especially reduction of disulfide bonds with DTT or β-ME^55^. We reasoned that keeping the dimeric structure intact might help us avoid all the extra bands in virus-treated cells that interfered with determining exactly where the reduced monomer might be otherwise located. Therefore, to keep CFTR in its dimeric state we did not boil the CFTR, and we did not add the reducing reagent DTT. Consistent with previous reports^55^, we were able to detect the physiological *ca.* 340 kDa CFTR dimer in a relatively band-free background (Fig. 8). As was the case for exposure of cells to spike proteins, Fig. 8 also showed that exposure of differentiated human lung epithelia to SARS-CoV-2 virus led to significant loss of CFTR dimer expression (*p* = 0.0169). Importantly, digitoxin and ouabain alone had little effect on CFTR expression. However, preincubation of virus with the cardiac glycoside drugs prevented SARS-CoV-2-dependent CFTR loss. These results underscore the significance of spike-dependent CFTR reduction following authentic virus infection, and highlight the potential of cardiac glycosides as possible treatments to prevent SARS-CoV-2 infection, CFTR loss and the subsequent inflammatory response.

## Discussion

The mechanism responsible for the proinflammatory phenotype of the COVID-19 airway remains to be fully elucidated^1–5^. Here, we have used a model of normal human small airway epithelium to demonstrate that SARS-CoV-2 spike-dependent loss of CFTR increases TNFα/NFκB signaling. Additionally, the spike protein-dependent loss of CFTR was accompanied by proteolytic activation of both α and γ ENaC. These two affected pathways are depicted schematically in Supplemental Fig. S1. Mechanistically, we found that ACE2 co-immunoprecipitated with CFTR, suggesting that ACE2 may be the physical link between spike protein and CFTR protein. Consistently, we found that the cardiac glycoside drugs ouabain, digitoxin and digoxin, which competitively inhibit binding of spike protein to ACE2^29^, were able to rescue spike-induced loss of cAMP-activated CFTR channel activity and CFTR protein expression. These results were recapitulated when differentiated lung epithelia were infected with authentic SARS-CoV-2 rather than exposed to pure spike proteins alone. Furthermore, we also found that loss of CFTR protein could be traced to inability to recover plasma membrane-localized CFTR from the endocytic recycling process. Since the purpose of endocytic recycling is to eliminate damaged proteins, it is possible that the spike protein, acting through ACE2, conferred some form of damage or instability on the CFTR. Finally, co-immunoprecipitation analyses suggested that the mechanism for spike-dependent elimination of CFTR might depend on spike first binding to ACE2. To our knowledge this is the first time COVID-19 airway inflammation has been experimentally linked to SARS-CoV-2 spike-dependent inhibition of CFTR signaling and recycling. This insight may have therapeutic implications since digitoxin is an inexpensive repurposed drug that has anti-inflammatory properties^34,35,56–58^, neutralizes SARS-CoV-2 through competitive inhibition of spike:ACE2 binding^29^, and, rescues CFTR protein from virus or spike protein-induced loss.

In support of possible therapeutic implications of the findings summarized above, a reasonable question might be to what extent a reduction in CFTR expression in the lung, or elsewhere in CF-affected tissues, might relate in any way to COVID-19 disease. One approach would be to consider the parents of children with CF. Since CF is an autosomal recessive disease, these parents, also termed CF carriers, each carry one mutant *CFTR* gene and one wildtype CFTR gene. The most common mutant CFTR genes, such as [*F508del]CFTR*, do not contribute significant amounts of functional CFTR protein^59^, and the remaining wildtype gene in CF carriers does not compensate for the loss of the mutant *CFTR* gene. Consequently, CF carriers suffer from reduction in CFTR function by approximately 50%^60–62^. Even under normal conditions these CF carriers have a high risk of CF-related comorbidity^63,64^. However, in data from the first pandemic wave in Italy, a cohort of hospitalized adult CF carriers with COVID-19 were found to be more likely than non-CF carriers to develop a form of COVID-19 characterized by acute respiratory distress syndrome, high inflammatory response and early mortality by day 14^65^. The analysis included an adjustment for age, sex and comorbidities. These results from CF carriers with COVID-19 have also been shown to have significant consequences on a global geographic scale of 37 countries, where CF carrier frequency was found to be directly correlated with COVID-19 spread and fatality^66^. Finally, CF carrier patients selected from the same first pandemic cohort in Italy, who had either Gain- or Loss-of-Function CFTR alleles, were recently shown to be associated with either mild or severe COVID-19, respectively^67^. These in vivo epidemiological data were validated by parallel functional studies in cultured cells bearing the same mutations. Thus, a reduction of CFTR function due to loss of the one normal CFTR gene in CF carriers would appear to be a potent risk factor for adverse outcomes in COVID-19 disease.

Nonetheless, early in the COVID-19 pandemic, it was widely noted that most CF patients, who lacked functional CFTR altogether, appeared to suffer only a mild course of COVID-19^37,68^. One possible explanation was that in the mutation-dependent absence of functional CFTR the spike protein receptor ACE2 was downregulated^39^. However, these data also showed that the extent of ACE2 downregulation in primary CF cells was not complete^39^. For example, analysis of primary cells from nasal and bronchial biopsies of CF patients left, respectively, 25% and 38% of ACE still expressed^39^. It was thus possible that the remaining ACE2 expression may contribute to the findings of a recent systematic review of nine observational studies that showed that there is a subgroup of people with CF that have a higher risk of severe outcome^68^. For this subgroup, risk factors included FEV1 < 70%predicted, CF-related diabetes, age > 40 Years, pancreatic insufficiency, underweight, previous transplant and azithromycin use^68^. In addition to the remaining ACE2 expression, another possible explanation for this heterogeneity in COVID-19 susceptibility could be that that independently of ACE2, the quality of life for people with cystic fibrosis may depend on contributions from non-CFTR modifier or bystander genes^69–75^. For example, we recently reported that Tensin 1 (*TNS1)* is a modifier gene for low body mass index (BMI) in homozygous [*F508del*]CFTR patients^76^. It is therefore possible that while CF carriers may not need the support of modifier genes for their personal survival in normal times, they may not easily tolerate further losses of CFTR expression following infection by SARS-CoV-2 virus. From this perspective, it may be that people with CF, who have appropriate modifier genes, may have a genetic propensity to survive without CFTR, and thus to also have lower risk of severe COVID-19. By contrast, CF carriers, who still express ACE2, but have only one normal CFTR gene, may therefore be more likely to have severe COVID than normal subjects who begin infection with normal levels of CFTR and ACE2.

The mechanisms remain to be fully determined by which (i) CFTR binds to ACE2 in non-CF lung epithelial cells, and by which (ii) CFTR is subsequently lost by endocytic recycling when spike protein or authentic virus binds to ACE2. The co-immunoprecipitation data presented here for differentiated BCi.NS1.1 cells, cultured 16HBE14o- cells, and differentiated primary HBE cells appear to be consistent with previously reported proximity ligation experiments on model non-CF lung epithelial cells such as CFBE41o- (WT) and 16HBE14o- cells^39^. By contrast, mutational loss of CFTR, either in cultured CF lung epithelial cells or in differentiated lung epithelia from CF patients, were associated with mislocalization of ACE2 away from the apical plasma membrane, thereby compromising infection by SARS-CoV-2^39^. Thus, the baseline situation in *people without CF*, the focus of this paper, may be fundamentally distinguished from the CF condition, in part, by the robust apical plasma membrane complex of CFTR with ACE2. It has been suggested that PDZ-interacting domains in ACE2^39,77–79^ and in CFTR^80–83^, might contribute to these interactions, either directly or indirectly through bridging proteins such as NHERF1 (sodium-hydrogen exchange regulatory factor 1)^39^. In ACE2, for example, elimination of the C-terminal PDZ domain results in reduced ACE2 membrane residence and concomitantly reduced viral entry^78^. Yet, there may be as many as 14 other PDZ binding proteins that can also bind to ACE2^79^, and the involvement of other PDZ-based linking proteins besides NHERF1 therefore cannot be excluded. In CFTR, the PDZ domains within CFTR itself have been known for decades to be critical for localization to the apical plasma membrane via interaction with NHERF1^80–83^. However, other PDZ-binding proteins are known to also regulate CFTR localization and function, including NHERF2^55,84^, CAL (CFTR-associated ligand)^85^, and CAP70 (CFTR associated protein)^86^ Furthermore, other non-PDZ-dependent mechanisms may contribute, since the physiologic dimers of CFTR are assembled independently of either NHERF1 or NHERF2, thus leaving PDZ-domains available for other interactions^55^. Thus, it is possible that other interactions could include binding to ACE2^39^. Finally, based on recent studies on the interaction of ACE2 and the SARS-CoV-2 virion, it is possible that the PDZ-based complex of ACE2, BAT^o^1 and β-arrestin^77^ may be mediated by NHERF1 to enable clathrin-assisted internalization into endosomes^78^. It is thus possible that such an endosomal recycling mechanism may also be responsible for the loss of CFTR that occurs after the spike protein or the intact virion binds to ACE2. However, while PDZ-domain interactions may contribute to CFTR-ACE2 binding and to spike protein- or virus- dependent loss of CFTR by endosomal recycling, the responsible mechanisms for these interactions remain to be determined.

In spite of the strong safety data from the Phase I/IIa digitoxin clinical trial on CF patients^87^, and the fact that COVID-19 is a lethal disease, drug safety does remain an issue for consideration. Fortunately, cardiac glycoside drugs have been in human use for centuries, and thus much is known about their pharmacokinetics and cytotoxicity^57^. For example, there are upper concentration limits when treating cardiac patients with these drugs who suffer from heart failure or arrhythmias^88^. However, for the “vast majority of patients with normal hearts, ingesting large but not lethal amounts of digitalis (meaning digitoxin or digoxin) either by accident or suicide attempt, seldom result in premature atrial contractions or irregular heartbeats”^88^. Consistently, no drug-related adverse events have been reported in trials on subjects with normal hearts who were given clinical doses of ouabain^89,90^, digitoxin^34,87^, or digoxin^91–94^. During the COVID-19 pandemic in the U.S., where only digoxin is available, we have recently reported that when heart failure patients with multiple comorbidities were treated with digoxin instead of standard-of-care drugs, both groups fared equivalently when faced with infection or survival with COVID-19^95^. Finally, a recent Phase I/Phase IIa clinical trial of digitoxin in adult people with cystic fibrosis showed that administering 0.1 mg digitoxin for 28 days met all indices for safety^87^. We therefore suggest that a potential safety concern for those with normal hearts should not preclude testing cardiac glycoside drugs such as digitoxin as a candidate therapeutic for COVID-19.

We can also consider whether data disclosed in this paper may also have importance for understanding and possibly treating Post-Acute Sequelae of COVID-19 (PASC) or “Long COVID”. It is of substantial public health concern is that up to 30% of acute COVID-19 patients may develop PASC, which can follow either a mild or severe infection^15,96^. Equally troubling are recent reports from a large Danish cohort that PASC can last for 12–18 months in 57% of patients, independent of viral strain^97^. However, it is presently not understood how PASC symptoms can be similar to that of the acute infection, in spite of the active infection having apparently terminated, and cryptic centers of infection being only infrequently found^16,98,99^. The problem is how such a systems-wide set of symptoms can be manifest for such a long time in the apparent absence of a system-wide cause. However, a possible solution may be found in reports of the occurrence of viral RNA and S1 spike derived protein fragments in circulating exosomes during acute infection^100,101^, and of isolated S1 (RBD, receptor binding domain) spike protein and nucleoprotein N in circulating PASC exosomes of neuronal origin^102^. Based on these data, exosomal spike proteins have been proposed to contribute to the COVID-19 adaptive immune response^100–102^. Thus, S1 spike protein, the part of the SARS-CoV-2 virus that directly contacts ACE2 in target tissues, and that induces loss of CFTR, may remain in production and distribution to the rest of the body long after the active infection phase has terminated^103,104^. Consistently, monoclonal antibodies against Spike protein have been suggested as possible therapeutics for long COVID^105^. It is therefore possible that exosomal S1 proteins in the circulation might contribute to system-wide PASC pathology. If this were the case, drugs like digitoxin, that blocked spike:ACE2 binding, should also be therapeutic for long COVID. This possibility may especially apply to those tissues in addition to lung, where ACE2 and CFTR are expressed, including heart^106^, kidney^107^ and brain^108,109^. Importantly, clinical doses of digitoxin and digoxin have been shown to penetrate into the CSF in normal humans^110^. It is therefore possible that cardiac glycosides such as digitoxin could also find a place for testing as therapeutics for long COVID/PASC.

Finally, it is important to mention here that induced losses of CFTR by environmental or infectious agents have been observed in the past. A widely described example is Chronic Obstructive Pulmonary Disease (COPD), which is associated with smoke-induced, sustained reduction in CFTR expression^111–114^. A second example is infection of HEK-293 cells by influenza strain A/Udorn, which has been reported to reduce CFTR chloride channel activity and CFTR protein^115,116^. The viral matrix protein M2 has been proposed to promote intracellular lysosomal degradation of CFTR, although the biochemical mechanism remains to be fully understood. Digitoxin has also been shown to block pulmonary inflammation, including TNFα and IFNγ, in a cotton rat model of Influenza A^117^. TNFα and IFNγ are of particular interest here because they may perpetuate cytokine storm in COVID-19^118^. An additional example is infection of BALB/c mice with mouse-adapted wildtype SARS-CoV virus, in which CFTR mRNA in lung is reduced by *ca* 60% on day 4 after infection^119^. Finally, CFTR expression in heart tissue has also been reported to be also downregulated in a mouse model of heart failure^109,120^. Thus multiple disease states have been described in which the CFTR protein can be diminished by an external factor rather than by a classical CFTR mutation.

This study has limitations. First, it is a limitation of our study that we do not yet understand how ACE2 binds to CFTR. Nonetheless, we now know (i) close association between the two proteins, in situ, has been recently described by others^39^; (ii) that the co-immunoprecipitation in normal lung epithelial cells is experimentally reciprocal; and (iii) that spike-dependent loss of apical CFTR is reflected by a loss of CFTR in the anti-ACE2 immunoprecipitate. The mechanism for increased ACE2 expression following spike-dependent loss of CFTR in normal lung epithelial cells is unknown, and thus remains a subject for future study. Second, it is a limitation that we do not yet understand the nature of the apparent damage or instability inflicted on CFTR by the [spike:ACE2:CFTR] interaction. Nonetheless, we do know that the exposure to spike protein enhances retention of CFTR in the endosomal compartment, indicating that damage has occurred. Third, it is a limitation that we have not proactively interrogated this mechanism using an in vivo model. However, we suggest that substantively addressing these questions would be clearly beyond the experimental scope of the present study and look forward to addressing them in future studies.

## Conclusion

Based on these investigations with a model of normal small airway epithelia and other normal lung epithelial cells, we predict that increased TNFα/NFκB- and ENaC- dependent inflammation in the COVID-19 airway is due to inhibition of CFTR signaling by SARS-CoV-2 spike protein, thus inducing a pro-inflammatory clinical phenotype in lung epithelia. Based on descriptions of more severe COVID-19 in adult CF carriers, who have only one copy of wildtype *CFTR*, we suggest that this model-based conclusion may be consistent with otherwise normal patient-based experience. To our knowledge this is the first time COVID-19 airway inflammation has been experimentally linked in normal subjects to SARS-CoV-2 spike-dependent inhibition of CFTR signaling. This insight may have therapeutic implications since digitoxin is an inexpensive repurposed drug that has anti-inflammatory properties^34,35,56–58^, neutralizes SARS-CoV-2 through competitive inhibition of spike:ACE2 binding^29^, and, rescues CFTR protein from virus or spike protein-induced loss.

## Methods

### Materials and reagents

Detailed materials and reagents are provided in the Supplemental Information S1.

### Cell cultures and treatments

We thank Dr. R.G. Crystal (Cornell Medical College, New York City, NY) for the gift of the hTERT-transformed BCi-NS1.1 basal stem cell. The basal cells were cultured, maintained, and differentiated on Transwell or Snapwell inserts under Air–Liquid Interface (ALI) conditions according to the instructions from Dr. Crystal’s lab. For spike protein treatment, differentiated BCi.NS1.1 (dBCi; ALI day 25–28) cells were exposed to different concentrations of [S1S2] spike protein on the apical/air side for 4 h, washed with PBS, and then incubated for additional 20 h at 37 °C and 5% CO_2_ under ALI conditions. For cardiac glycoside treatment, spike protein was preincubated with cardiac glycosides for 1 h at 37 °C prior to addition to cells on the apical side, followed by washing and further incubation for additional 20 h under ALI conditions. The human bronchial epithelial cell line 16HBE14o- was purchased from Sigma-Aldrich (#SCC150) and cultured according to the manufacture instructions. Submerged 16HBE14o- cell cultures were treated with spike protein in the presence or absence of cardiac glycoside drugs for 4 h in Serum-free MEM medium, washed out, and then incubated in the complete MEM medium for additional 20 h.

Authentic SARS-CoV-2 β virus was propagated in Vero E6 cells and used to infect dBCi cell monolayers. Prior to infection, titrated virus (200,000 PFU/12-mm well) was mixed with ALI growth medium containing 50 nM digitoxin or ouabain for 1 h at 37 °C. Cell monolayers were infected apically with medium, digitoxin, ouabain, SARS-CoV-2, or SARS-CoV-2 + digitoxin or ouabain for 4 h. Cells were washed twice with PBS and incubated for additional 20 h under ALI conditions (see Supplemental Information S1 for more details).

### Ussing chamber analysis

Cyclic AMP-activated CFTR chloride channel activity of dBCi cell monolayers was measured using a Ussing chamber as previously described^34^. The method is detailed in the Supplemental Information S1.

### Cell surface biotinylation and endosomal recycling assay

Surface biotinylation and endosomal recycling was performed as described previously^49^. For surface biotinylation, plasma membrane proteins of spike-treated dBCi cell monolayers were biotinylated at 4 °C using membrane-impermeable and cleavable EZ-Link™ Sulfo-NHS-SS-Biotin. For the endosomal recycling assay, dBCi cell monolayers were first biotinylated and then incubated at 37 °C for 4 h with spike protein apically, or additional 20 h without Spike protein under ALI conditions. Subsequently, the disulfide bonds on Sulfo-NHS-SS-biotinylated proteins remaining at the plasma membrane were stripped with L-glutathione (GSH) at 4 °C. At this point of the protocol, biotinylated proteins residing within the endosomal compartment were protected from GSH. Biotinylated dBCi cell monolayers, either treated with or without GSH, were used as controls for both GSH treatment and biotinylation processes, respectively. Biotinylated CFTR was isolated with streptavidin-agarose beads and analyzed by western blotting.

### Co-immunoprecipitation of ACE2 and CFTR

Total lysates of untreated basal or differentiated BCi.NS1.1 cells were precleared with Protein A Dynabeads, followed by incubation for 24 h at 4 °C with 2 μg of normal rabbit serum or anti-ACE2 antibody, washing 3 times with RIPA buffer, and incubation for 4 h with Protein A Dynabeads. CFTR in the immunoprecipitated complexes was analyzed by western blotting.

### Western blotting

Treated cells were washed with PBS and lysed in RIPA buffer supplemented with the anti-protease/phosphatase cocktail. Equivalent amounts of lysates (50 μg/sample) were electrophoresed on 4–12% or 4–20% gradient gels (Invitrogen), transferred to PVDF membranes, and membranes were probed with β-Actin antibody (Sigma) or CFTR antibody combo (UNC 450, UNC596 and UNC570) at 1:1000 dilution each. For Western blotting analyses of proteins in the NFκB signaling pathway, including ENaC, TRADD or TNFR1, the antibodies were diluted and used according to the manufacture’s recommended instructions. Immunoreactive bands were visualized using BioRad ChemiDocTM Imaging System and quantified using the Imagelab software. A more detailed description for Western blot with authentic SARS-CoV-2 virus is given in the Supplement.

### Measurement of IL-8

Culture media from the basolateral compartments were collected and used to assay for IL-8 using a DuoSet® ELISA kit obtained from R&D Systems, and performed according to the manufacturer's instructions.

### Statistics

Statistical *p* values were determined with a one-way ANOVA, followed either by Holm’s and/or Dunnett’s post-hoc tests as specified in Figure legends.

### Supplementary Information

Supplementary Information.

## Data Availability

The datasets generated and/or analyzed during the current study are available from the corresponding author on reasonable request. The opinions, interpretations, conclusions and recommendations are those of the authors and are not necessarily endorsed by the U.S. Army, Department of Defense, the U.S. Government or the Uniformed Services University of the Health Sciences. The use of trade names does not constitute an official endorsement or approval of the use of such reagents or commercial hardware or software. This document may not be cited for purposes of advertisement.

## References

[1] Chen, G. *et al.* Clinical and immunological features of severe and moderate coronavirus disease 2019. *J. Clin. Investig.* **130**, 2620–2629. 10.1172/jci137244 (2020). 32217835 10.1172/jci137244 PMC7190990

[2] Zhou, Z. *et al.* Heightened innate immune responses in the respiratory tract of COVID-19 patients. *Cell Host Microbe* **27**, 883-890.e882. 10.1016/j.chom.2020.04.017 (2020). 32407669 10.1016/j.chom.2020.04.017 PMC7196896

[3] Huang, C. *et al.* Clinical features of patients infected with 2019 novel coronavirus in Wuhan China. *Lancet* **395**, 497–506. 10.1016/s0140-6736(20)30183-5 (2020). 31986264 10.1016/s0140-6736(20)30183-5 PMC7159299

[4] Tisoncik, J. R. *et al.* Into the eye of the cytokine storm. *Microbiol. Mol. Biol. Rev.* **76**, 16–32. 10.1128/mmbr.05015-11 (2012). 22390970 10.1128/mmbr.05015-11 PMC3294426

[5] Channappanavar, R. & Perlman, S. Pathogenic human coronavirus infections: Causes and consequences of cytokine storm and immunopathology. *Semin. Immunopathol.* **39**, 529–539. 10.1007/s00281-017-0629-x (2017). 28466096 10.1007/s00281-017-0629-x PMC7079893

[6] DeDiego, M. L. *et al.* Inhibition of NF-κB-mediated inflammation in severe acute respiratory syndrome coronavirus-infected mice increases survival. *J. Virol.* **88**, 913–924. 10.1128/jvi.02576-13 (2014). 24198408 10.1128/jvi.02576-13 PMC3911641

[7] Su, C. M., Wang, L. & Yoo, D. Activation of NF-κB and induction of proinflammatory cytokine expressions mediated by ORF7a protein of SARS-CoV-2. *Sci. Rep.* **11**, 13464. 10.1038/s41598-021-92941-2 (2021). 34188167 10.1038/s41598-021-92941-2 PMC8242070

[8] Anand, P., Puranik, A., Aravamudan, M., Venkatakrishnan, A. J. & Soundararajan, V. SARS-CoV-2 strategically mimics proteolytic activation of human ENaC. *eLife* **9**. 10.7554/eLife.58603 (2020). 10.7554/eLife.58603 PMC7343387 32452762

[9] Gentzsch, M. & Rossier, B. C. A Pathophysiological model for COVID-19: Critical importance of transepithelial sodium transport upon airway infection. *Function (Oxf)* **1**, zqaa024. 10.1093/function/zqaa024 (2020). 10.1093/function/zqaa024 PMC7662147 33201937

[10] Zhang, Q., Lenardo, M. J. & Baltimore, D. 30 years of NF-kappaB: A blossoming of relevance to human pathobiology. *Cell* **168**, 37–57. 10.1016/j.cell.2016.12.012 (2017). 28086098 10.1016/j.cell.2016.12.012 PMC5268070

[11] Reusch, N. *et al.* Neutrophils in COVID-19. *Front. Immunol.* **12**, 652470. 10.3389/fimmu.2021.652470 (2021). 33841435 10.3389/fimmu.2021.652470 PMC8027077

[12] Chua, R. L. *et al.* COVID-19 severity correlates with airway epithelium-immune cell interactions identified by single-cell analysis. *Nat. Biotechnol.* **38**, 970–979. 10.1038/s41587-020-0602-4 (2020). 32591762 10.1038/s41587-020-0602-4

[13] Hobbs, C. A. *et al.* Identification of the SPLUNC1 ENaC-inhibitory domain yields novel strategies to treat sodium hyperabsorption in cystic fibrosis airway epithelial cultures. *Am. J. Physiol. Lung Cell. Mol. Physiol.* **305**, L990–L1001. 10.1152/ajplung.00103.2013 (2013). 10.1152/ajplung.00103.2013 PMC3882538 24124190

[14] Mall, M. A. ENaC inhibition in cystic fibrosis: potential role in the new era of CFTR modulator therapies. *Eur. Respir. J.* **56**, 1. 10.1183/13993003.00946-2020 (2020). 10.1183/13993003.00946-2020 PMC7758539 32732328

[15] Proal, A. D. & VanElzakker, M. B. Long COVID or post-acute Sequelae of COVID-19 (PASC): An overview of biological factors that may contribute to persistent symptoms. *Front. Microbiol.* **12**, 698169. 10.3389/fmicb.2021.698169 (2021). 34248921 10.3389/fmicb.2021.698169 PMC8260991

[16] Chertow, D. *et al.* SARS-CoV-2 infection and persistence throughout the human body and brain. *Res. Square*. 10.21203/rs.3.rs-1139035/v1 (2021).

[17] Al-Aly, Z., Xie, Y. & Bowe, B. High-dimensional characterization of post-acute sequelae of COVID-19. *Nature* **594**, 259–264. 10.1038/s41586-021-03553-9 (2021). 33887749 10.1038/s41586-021-03553-9

[18] Vabret, N. *et al.* Immunology of COVID-19: Current State of the Science. *Immunity* **52**, 910–941. 10.1016/j.immuni.2020.05.002 (2020). 32505227 10.1016/j.immuni.2020.05.002 PMC7200337

[19] Neufeldt, C. J. *et al.* SARS-CoV-2 infection induces a pro-inflammatory cytokine response through cGAS-STING and NF-κB. *Commun. Biol.* **5**, 45. 10.1038/s42003-021-02983-5 (2022). 35022513 10.1038/s42003-021-02983-5 PMC8755718

[20] Diamond, M. S. & Kanneganti, T. D. Innate immunity: The first line of defense against SARS-CoV-2. *Nat. Immunol.* **23**, 165–176. 10.1038/s41590-021-01091-0 (2022). 35105981 10.1038/s41590-021-01091-0 PMC8935980

[21] Su, J. *et al.* SARS-CoV-2 ORF3a inhibits cGAS-STING-mediated autophagy flux and antiviral function. *J. Med. Virol.* **95**, e28175. 10.1002/jmv.28175 (2023). 36163413 10.1002/jmv.28175 PMC9538343

[22] Karki, R. & Kanneganti, T. D. Innate immunity, cytokine storm, and inflammatory cell death in COVID-19. *J. Transl. Med.* **20**, 542. 10.1186/s12967-022-03767-z (2022). 36419185 10.1186/s12967-022-03767-z PMC9682745

[23] Bouvet, M. *et al.* In vitro reconstitution of SARS-coronavirus mRNA cap methylation. *PLoS Pathogens* **6**, e1000863. 10.1371/journal.ppat.1000863 (2010). 20421945 10.1371/journal.ppat.1000863 PMC2858705

[24] Deng, X. *et al.* Coronavirus nonstructural protein 15 mediates evasion of dsRNA sensors and limits apoptosis in macrophages. *Proc. Natl. Acad. Sci. USA* **114**, E4251-e4260. 10.1073/pnas.1618310114 (2017). 28484023 10.1073/pnas.1618310114 PMC5448190

[25] Hackbart, M., Deng, X. & Baker, S. C. Coronavirus endoribonuclease targets viral polyuridine sequences to evade activating host sensors. *Proc. Natl. Acad. Sci. USA* **117**, 8094–8103. 10.1073/pnas.1921485117 (2020). 32198201 10.1073/pnas.1921485117 PMC7149396

[26] Ivanov, K. A. *et al.* Multiple enzymatic activities associated with severe acute respiratory syndrome coronavirus helicase. *J. Virol.* **78**, 5619–5632. 10.1128/jvi.78.11.5619-5632.2004 (2004). 15140959 10.1128/jvi.78.11.5619-5632.2004 PMC415832

[27] Knoops, K. *et al.* SARS-coronavirus replication is supported by a reticulovesicular network of modified endoplasmic reticulum. *PLoS Biol.* **6**, e226. 10.1371/journal.pbio.0060226 (2008). 18798692 10.1371/journal.pbio.0060226 PMC2535663

[28] Li, J. Y. *et al.* The ORF6, ORF8 and nucleocapsid proteins of SARS-CoV-2 inhibit type I interferon signaling pathway. *Virus Res.* **286**, 198074. 10.1016/j.virusres.2020.198074 (2020). 32589897 10.1016/j.virusres.2020.198074 PMC7309931

[29] Caohuy, H. *et al.* Common cardiac medications potently inhibit ACE2 binding to the SARS-CoV-2 Spike, and block virus penetration and infectivity in human lung cells. *Sci. Rep.* **11**, 22195. 10.1038/s41598-021-01690-9 (2021). 34773067 10.1038/s41598-021-01690-9 PMC8589851

[30] Cho, J. *et al.* Antiviral activity of digoxin and ouabain against SARS-CoV-2 infection and its implication for COVID-19. *Sci. Rep.* **10**, 16200. 10.1038/s41598-020-72879-7 (2020). 33004837 10.1038/s41598-020-72879-7 PMC7530981

[31] Berdiev, B. K., Qadri, Y. J. & Benos, D. J. Assessment of the CFTR and ENaC association. *Mol. Biosyst.* **5**, 123–127. 10.1039/b810471a (2009). 19156256 10.1039/b810471a PMC2666849

[32] Abdel Hameid, R., Cormet-Boyaka, E., Kuebler, W. M., Uddin, M. & Berdiev, B. K. SARS-CoV-2 may hijack GPCR signaling pathways to dysregulate lung ion and fluid transport. *Am. J. Physiol. Lung Cell. Mol. Physiol.* **320**, L430–L435. 10.1152/ajplung.00499.2020 (2021). 10.1152/ajplung.00499.2020 PMC7938641 33434105

[33] Wang, H. *et al.* CFTR controls the activity of NF-kappaB by enhancing the degradation of TRADD. *Cell. Physiol. Biochem.* **40**, 1063–1078. 10.1159/000453162 (2016). 27960153 10.1159/000453162 PMC7067292

[34] Yang, Q. *et al.* Gene therapy-emulating small molecule treatments in cystic fibrosis airway epithelial cells and patients. *Respir. Res.* **20**, 290. 10.1186/s12931-019-1214-8 (2019). 31864360 10.1186/s12931-019-1214-8 PMC6925517

[35] Srivastava, M. *et al.* Digitoxin mimics gene therapy with CFTR and suppresses hypersecretion of IL-8 from cystic fibrosis lung epithelial cells. *Proc. Natl. Acad. Sci. USA* **101**, 7693–7698. 10.1073/pnas.0402030101 (2004). 15136726 10.1073/pnas.0402030101 PMC419668

[36] Stanton, B. A., Hampton, T. H. & Ashare, A. SARS-CoV-2 (COVID-19) and cystic fibrosis. *Am. J. Physiol. Lung Cell. Mol. Physiol.* **319**, L408-L415. 10.1152/ajplung.00225.2020 (2020). 10.1152/ajplung.00225.2020 PMC7518058 32668165

[37] Peckham, D., McDermott, M. F., Savic, S. & Mehta, A. COVID-19 meets Cystic Fibrosis: For better or worse?. *Genes Immunity* **21**, 260–262. 10.1038/s41435-020-0103-y (2020). 32606316 10.1038/s41435-020-0103-y

[38] Scambler, T. *et al.* ENaC-mediated sodium influx exacerbates NLRP3-dependent inflammation in cystic fibrosis. *eLife* **8**. 10.7554/eLife.49248 (2019). 10.7554/eLife.49248 PMC6764826 31532390

[39] Bezzerri, V. *et al.* SARS-CoV-2 viral entry and replication is impaired in Cystic Fibrosis airways due to ACE2 downregulation. *Nat. Commun.* **14**, 132. 10.1038/s41467-023-35862-0 (2023). 36627352 10.1038/s41467-023-35862-0 PMC9830623

[40] Caohuy, H. *et al.* Inflammation in the COVID-19 airway is due to inhibition of CFTR signaling by the SARS-CoV-2 Spike protein. *bioRxiv*. 10.1101/2022.01.18.476803 (2022). 10.1038/s41598-024-66473-4 PMC11266487 39043712

[41] Walters, M. S. *et al.* Generation of a human airway epithelium derived basal cell line with multipotent differentiation capacity. *Respir. Res.* **14**, 135. 10.1186/1465-9921-14-135 (2013). 24298994 10.1186/1465-9921-14-135 PMC3907041

[42] Wang, G. *et al.* Characterization of an immortalized human small airway basal stem/progenitor cell line with airway region-specific differentiation capacity. *Respir. Res.* **20**, 196. 10.1186/s12931-019-1140-9 (2019). 31443657 10.1186/s12931-019-1140-9 PMC6708250

[43] Zhang, H. *et al.* Expression of the SARS-CoV-2 ACE2 Receptor in the Human Airway Epithelium. *Am. J. Respir. Crit. Care Med.* **202**, 219–229. 10.1164/rccm.202003-0541OC (2020). 32432483 10.1164/rccm.202003-0541OC PMC7365377

[44] Schoumacher, R. A. *et al.* A cystic fibrosis pancreatic adenocarcinoma cell line. *Proc. Natl. Acad. Sci. USA* **87**, 4012–4016 (1990). 1692630 10.1073/pnas.87.10.4012 PMC54034

[45] Caohuy, H. *et al.* Activation of 3-phosphoinositide-dependent kinase 1 (PDK1) and serum- and glucocorticoid-induced protein kinase 1 (SGK1) by short-chain sphingolipid C4-ceramide rescues the trafficking defect of DeltaF508-cystic fibrosis transmembrane conductance regulator (DeltaF508-CFTR). *J. Biol. Chem.* **289**, 35953–35968. 10.1074/jbc.M114.598649 (2014). 25384981 10.1074/jbc.M114.598649 PMC4276863

[46] Zhang, L. *et al.* CFTR delivery to 25% of surface epithelial cells restores normal rates of mucus transport to human cystic fibrosis airway epithelium. *PLoS Biol.* **7**, e1000155. 10.1371/journal.pbio.1000155 (2009). 19621064 10.1371/journal.pbio.1000155 PMC2705187

[47] Sharma, M. *et al.* Misfolding diverts CFTR from recycling to degradation: quality control at early endosomes. *J. Cell Biol.* **164**, 923–933. 10.1083/jcb.200312018 (2004). 15007060 10.1083/jcb.200312018 PMC2172283

[48] Ameen, N., Silvis, M. & Bradbury, N. A. Endocytic trafficking of CFTR in health and disease. *J. Cyst. Fibrosis.* **6**, 1–14. 10.1016/j.jcf.2006.09.002 (2007). 10.1016/j.jcf.2006.09.002 PMC1964799 17098482

[49] Bomberger, J. M., Guggino, W. B. & Stanton, B. A. Methods to monitor cell surface expression and endocytic trafficking of CFTR in polarized epithelial cells. *Methods Mol. Biol. (Clifton, N.J.)* **741**, 271–283. 10.1007/978-1-61779-117-8_18 (2011). 10.1007/978-1-61779-117-8_18 PMC4402161 21594791

[50] García-Caballero, A., Dang, Y., He, H. & Stutts, M. J. ENaC proteolytic regulation by channel-activating protease 2. *J. Gen. Physiol.* **132**, 521–535. 10.1085/jgp.200810030 (2008). 18852303 10.1085/jgp.200810030 PMC2571966

[51] Haerteis, S. *et al.* Proteolytic activation of the epithelial sodium channel (ENaC) by the cysteine protease cathepsin-S. *Pflugers Archiv Eur. J. Physiol.* **464**, 353–365. 10.1007/s00424-012-1138-3 (2012). 22864553 10.1007/s00424-012-1138-3 PMC3448907

[52] Schillers, H., Shahin, V., Albermann, L., Schafer, C. & Oberleithner, H. Imaging CFTR: A tail to tail dimer with a central pore. *Cell. Physiol. Biochem. Int. J. Exp. Cell. Physiol. Biochem. Pharmacol.* **14**, 1–10. 10.1159/000076921 (2004). 10.1159/000076921 14976401

[53] Schillers, H. Imaging CFTR in its native environment. *Pflugers Archiv. Eur. J. Physiol.* **456**, 163–177. 10.1007/s00424-007-0399-8 (2008). 18057957 10.1007/s00424-007-0399-8

[54] Zhang, L., Aleksandrov, L. A., Riordan, J. R. & Ford, R. C. Domain location within the cystic fibrosis transmembrane conductance regulator protein investigated by electron microscopy and gold labelling. *Biochim. Biophys. Acta* **1808**, 399–404. 10.1016/j.bbamem.2010.08.012 (2011). 20727849 10.1016/j.bbamem.2010.08.012

[55] Li, C., Roy, K., Dandridge, K. & Naren, A. P. Molecular assembly of cystic fibrosis transmembrane conductance regulator in plasma membrane. *J. Biol. Chem.* **279**, 24673–24684. 10.1074/jbc.M400688200 (2004). 15060073 10.1074/jbc.M400688200

[56] Yang, Q. *et al.* Cardiac glycosides inhibit TNF-alpha/NF-kappaB signaling by blocking recruitment of TNF receptor-associated death domain to the TNF receptor. *Proc. Natl. Acad. Sci. USA* **102**, 9631–9636. 10.1073/pnas.0504097102 (2005). 15983368 10.1073/pnas.0504097102 PMC1160519

[57] Pollard, B. S., Blanco, J. C. & Pollard, J. R. Classical drug digitoxin inhibits influenza cytokine storm, with implications for covid-19 therapy. *In vivo (Athens, Greece)* **34**, 3723–3730. 10.21873/invivo.12221 (2020). 10.21873/invivo.12221 PMC7811644 33144490

[58] Miller, S. C. *et al.* Identification of known drugs that act as inhibitors of NF-kappaB signaling and their mechanism of action. *Biochem. Pharmacol.* **79**, 1272–1280. 10.1016/j.bcp.2009.12.021 (2010). 20067776 10.1016/j.bcp.2009.12.021 PMC2834878

[59] Veit, G. *et al.* From CFTR biology toward combinatorial pharmacotherapy: Expanded classification of cystic fibrosis mutations. *Mol. Biol. Cell* **27**, 424–433. 10.1091/mbc.E14-04-0935 (2016). 26823392 10.1091/mbc.E14-04-0935 PMC4751594

[60] Gabriel, S. E., Brigman, K. N., Koller, B. H., Boucher, R. C. & Stutts, M. J. Cystic fibrosis heterozygote resistance to cholera toxin in the cystic fibrosis mouse model. *Science (New York, N.Y.)* **266**, 107–109. 10.1126/science.7524148 (1994). 10.1126/science.7524148 7524148

[61] Trapnell, B. C. *et al.* Expression of the cystic fibrosis transmembrane conductance regulator gene in the respiratory tract of normal individuals and individuals with cystic fibrosis. *Proc. Natl. Acad. Sci. USA* **88**, 6565–6569. 10.1073/pnas.88.15.6565 (1991). 1713683 10.1073/pnas.88.15.6565 PMC52127

[62] Wine, J. J. How the sweat gland reveals levels of CFTR activity. *J. Cyst. Fibrosis.* **21**, 396–406. 10.1016/j.jcf.2022.02.001 (2022). 10.1016/j.jcf.2022.02.001 35184981

[63] Çolak, Y., Nordestgaard, B. G. & Afzal, S. Morbidity and mortality in carriers of the cystic fibrosis mutation CFTR Phe508del in the general population. *Eur. Respir. J.* **56**, 1. 10.1183/13993003.00558-2020 (2020). 10.1183/13993003.00558-2020 32398304

[64] Miller, A. C. *et al.* Cystic fibrosis carriers are at increased risk for a wide range of cystic fibrosis-related conditions. *Proc. Natl. Acad. Sci. USA* **117**, 1621–1627. 10.1073/pnas.1914912117 (2020). 31882447 10.1073/pnas.1914912117 PMC6983448

[65] Baldassarri, M. *et al.* Severe COVID-19 in hospitalized carriers of single CFTR pathogenic variants. *J. Pers. Med.* **11**. 10.3390/jpm11060558 (2021). 10.3390/jpm11060558 PMC8232773 34203982

[66] Gabbi, C., Renieri, A. & Strandvik, B. Geographical distribution of cystic fibrosis carriers as population genetic determinant of COVID-19 spread and fatality in 37 countries. *J. Infect.*10.1016/j.jinf.2022.06.006 (2022). 35700866 10.1016/j.jinf.2022.06.006 PMC9188282

[67] Baldassarri, M. *et al.* Gain- and Loss-of-function CFTR alleles are associated with COVID-19 clinical outcomes. *Cells* **11**. 10.3390/cells11244096 (2022). 10.3390/cells11244096 PMC9776607 36552859

[68] Terlizzi, V., Motisi, M. A., Pellegrino, R., Padoan, R. & Chiappini, E. Risk factors for severe COVID-19 in people with cystic fibrosis: A systematic review. *Front. Pediatr.* **10**, 958658. 10.3389/fped.2022.958658 (2022). 36003489 10.3389/fped.2022.958658 PMC9393295

[69] Guillot, L. *et al.* Lung disease modifier genes in cystic fibrosis. *Int. J. Biochem. Cell Biol.* **52**, 83–93. 10.1016/j.biocel.2014.02.011 (2014). 24569122 10.1016/j.biocel.2014.02.011

[70] Beucher, J. *et al.* AGER -429T/C is associated with an increased lung disease severity in cystic fibrosis. *PloS one* **7**, e41913. 10.1371/journal.pone.0041913 (2012). 22860029 10.1371/journal.pone.0041913 PMC3408394

[71] Drumm, M. L. *et al.* Genetic modifiers of lung disease in cystic fibrosis. *N. Engl. J. Med.* **353**, 1443–1453. 10.1056/NEJMoa051469 (2005). 16207846 10.1056/NEJMoa051469

[72] Marson, F. A., Bertuzzo, C. S., Ribeiro, A. F. & Ribeiro, J. D. Polymorphisms in the glutathione pathway modulate cystic fibrosis severity: A cross-sectional study. *BMC Med. Genet.* **15**, 27. 10.1186/1471-2350-15-27 (2014). 24593045 10.1186/1471-2350-15-27 PMC3973994

[73] Coutinho, C. A. *et al.* TNF-alpha polymorphisms as a potential modifier gene in the cystic fibrosis. *Int. J. Mol. Epidemiol. Genet.* **5**, 87–99 (2014). 24959313 PMC4065397

[74] Furlan, L. L. *et al.* IL8 gene as modifier of cystic fibrosis: Unraveling the factors which influence clinical variability. *Hum. Genet.* **135**, 881–894. 10.1007/s00439-016-1684-4 (2016). 27209008 10.1007/s00439-016-1684-4

[75] Cutting, G. R. Modifier genes in Mendelian disorders: The example of cystic fibrosis. *Ann. N. Y. Acad. Sci.* **1214**, 57–69. 10.1111/j.1749-6632.2010.05879.x (2010). 21175684 10.1111/j.1749-6632.2010.05879.x PMC3040597

[76] Walton, N. I. *et al.* Tensin 1 (TNS1) is a modifier gene for low body mass index (BMI) in homozygous [F508del]CFTR patients. *Physiol. Rep.* **9**, e14886. 10.14814/phy2.14886 (2021). 10.14814/phy2.14886 PMC8176904 34086412

[77] Yan, R. *et al.* Structural basis for the recognition of SARS-CoV-2 by full-length human ACE2. *Science (New York, N.Y.)* **367**, 1444–1448. 10.1126/science.abb2762 (2020). 10.1126/science.abb2762 PMC7164635 32132184

[78] Zhang, Q., Gefter, J., Sneddon, W. B., Mamonova, T. & Friedman, P. A. ACE2 interaction with cytoplasmic PDZ protein enhances SARS-CoV-2 invasion. *iScience* **24**, 102770. 10.1016/j.isci.2021.102770 (2021). 10.1016/j.isci.2021.102770 PMC8223119 34189428

[79] Caillet-Saguy, C. & Wolff, N. PDZ-containing proteins targeted by the ACE2 receptor. *Viruses* **13**. 10.3390/v13112281 (2021). 10.3390/v13112281 PMC8624105 34835087

[80] Hall, R. A. *et al.* A C-terminal motif found in the beta2-adrenergic receptor, P2Y1 receptor and cystic fibrosis transmembrane conductance regulator determines binding to the Na+/H+ exchanger regulatory factor family of PDZ proteins. *Proc. Natl. Acad. Sci. USA* **95**, 8496–8501. 10.1073/pnas.95.15.8496 (1998). 9671706 10.1073/pnas.95.15.8496 PMC21104

[81] Wang, S., Raab, R. W., Schatz, P. J., Guggino, W. B. & Li, M. Peptide binding consensus of the NHE-RF-PDZ1 domain matches the C-terminal sequence of cystic fibrosis transmembrane conductance regulator (CFTR). *FEBS Lett.* **427**, 103–108. 10.1016/s0014-5793(98)00402-5 (1998). 9613608 10.1016/s0014-5793(98)00402-5

[82] Benharouga, M. *et al.* The role of the C terminus and Na+/H+ exchanger regulatory factor in the functional expression of cystic fibrosis transmembrane conductance regulator in nonpolarized cells and epithelia. *J. Biol. Chem.* **278**, 22079–22089. 10.1074/jbc.M301030200 (2003). 12651858 10.1074/jbc.M301030200

[83] Kwon, S. H., Pollard, H. & Guggino, W. B. Knockdown of NHERF1 enhances degradation of temperature rescued DeltaF508 CFTR from the cell surface of human airway cells. *Cell. Physiol. Biochem. Int. J. Exp. Cell. Physiol. Biochem. Pharmacol.* **20**, 763–772. 10.1159/000110436 (2007). 10.1159/000110436 17982258

[84] Zhang, W., Zhang, Z., Zhang, Y. & Naren, A. P. CFTR-NHERF2-LPA₂ complex in the airway and gut epithelia. *Int. J. Mol. Sci.* **18**. 10.3390/ijms18091896 (2017). 10.3390/ijms18091896 PMC5618545 28869532

[85] Naren, A. P. *et al.* A macromolecular complex of beta 2 adrenergic receptor, CFTR, and ezrin/radixin/moesin-binding phosphoprotein 50 is regulated by PKA. *Proc. Natl. Acad. Sci. USA* **100**, 342–346. 10.1073/pnas.0135434100 (2003). 12502786 10.1073/pnas.0135434100 PMC140971

[86] Wang, S., Yue, H., Derin, R. B., Guggino, W. B. & Li, M. Accessory protein facilitated CFTR-CFTR interaction, a molecular mechanism to potentiate the chloride channel activity. *Cell* **103**, 169–179. 10.1016/s0092-8674(00)00096-9 (2000). 11051556 10.1016/s0092-8674(00)00096-9

[87] Zeitlin, P. L. *et al.* Digitoxin for airway inflammation in cystic fibrosis: Preliminary assessment of safety, pharmacokinetics, and dose finding. *Ann. Am. Thorac. Soc.* **14**, 220–229. 10.1513/AnnalsATS.201608-649OC (2017). 28006108 10.1513/AnnalsATS.201608-649OC PMC5427734

[88] Hoffman, B. J. & Bigger, J. *Digitalis and Allied Cardiac Glycosides Goodman and Gilman's The Pharmacological Basis of Therapeutics* Eith Edition edn, 833 (Permagon Press, 1990).

[89] Mason, D. T. & Braunwald, E. Studies on digitalis. X. Effects of ouabain on forearm vascular resistance and venous tone in normal subjects and in patients in heart failure. *J. Clin. Investig.* **43**, 532–543. 10.1172/jci104939 (1964). 14135505 10.1172/jci104939 PMC441947

[90] Selden, R. & Smith, T. W. Ouabain pharmacokinetics in dog and man. *Determination by radioimmunoassay. Circulation* **45**, 1176–1182. 10.1161/01.cir.45.6.1176 (1972). 5032817 10.1161/01.cir.45.6.1176

[91] Coates, A. L., Desmond, K., Asher, M. I., Hortop, J. & Beaudry, P. H. The effect of digoxin on exercise capacity and exercising cardiac function in cystic fibrosis. *Chest* **82**, 543–547 (1982). 7128221 10.1378/chest.82.5.543

[92] Moss, A. J. *et al.* Absorption of digoxin in children with cystic fibrosis. *J. Pediatr.* **86**, 295–297 (1975). 1111698 10.1016/S0022-3476(75)80493-8

[93] Selzer, A., Hultgren, H. N., Ebnother, C. L., Bradley, H. W. & Stone, A. O. Efect of digoxin on the circulation in normal man. *Br. Heart J.* **21**, 335–342. 10.1136/hrt.21.3.335 (1959). 14444797 10.1136/hrt.21.3.335 PMC1017589

[94] Williams, M. H. Jr., Zohman, L. R. & Ratner, A. C. Hemodynamic effects of cardiac glycosides on normal human subjects during rest and exercise. *J. Appl. Physiol.* **13**, 417–421. 10.1152/jappl.1958.13.3.417 (1958). 13587425 10.1152/jappl.1958.13.3.417

[95] Banaag, A. L., Pollard, H. B. & Koehlmoos, T. P. Digoxin and standard-of-care therapy for heart failure patients with COVID-19: Analysis of data from the US military health system (MHS) data repository. *Drugs Real World Outcomes* 1–9. 10.1007/s40801-023-00360-8 (2023). 10.1007/s40801-023-00360-8 PMC10024520 36933173

[96] Yoo, S. M. *et al.* Factors associated with post-acute sequelae of SARS-CoV-2 (PASC) after diagnosis of symptomatic COVID-19 in the inpatient and outpatient setting in a diverse cohort. *J. Gen. Intern. Med*. 1–8. 10.1007/s11606-022-07523-3 (2022). 10.1007/s11606-022-07523-3 PMC8989256 35391623

[97] Agergaard, J., Gunst, J. D., Schiøttz-Christensen, B., Østergaard, L. & Wejse, C. Long-term Prognosis at 1.5 years after infection with wild-type strain of SARS-CoV-2 and Alpha, Delta, as well as omicron variants. *Int. J. Infect. Dis.*. 10.1016/j.ijid.2023.10.022 (2023). 10.1016/j.ijid.2023.10.022 37907167

[98] Davis, H. E., McCorkell, L., Vogel, J. M. & Topol, E. J. Long COVID: Major findings, mechanisms and recommendations. *Nat. Rev. Microbiol.* **21**, 133–146. 10.1038/s41579-022-00846-2 (2023). 36639608 10.1038/s41579-022-00846-2 PMC9839201

[99] Bajema, K. L. *et al.* Effectiveness of COVID-19 treatment with nirmatrelvir-ritonavir or molnupiravir among US veterans: Target trial emulation studies with one-month and six-month outcomes. *Ann. Intern. Med.* **176**, 807–816. 10.7326/m22-3565 (2023). 37276589 10.7326/m22-3565 PMC10243488

[100] Barberis, E. *et al.* Circulating exosomes are strongly involved in SARS-CoV-2 infection. *Front. Mol. Biosci.* **8**, 632290. 10.3389/fmolb.2021.632290 (2021). 33693030 10.3389/fmolb.2021.632290 PMC7937875

[101] Pesce, E. *et al.* Exosomes recovered from the plasma of COVID-19 patients expose SARS-CoV-2 spike-derived fragments and contribute to the adaptive immune response. *Front. Immunol.* **12**, 785941. 10.3389/fimmu.2021.785941 (2021). 35111156 10.3389/fimmu.2021.785941 PMC8801440

[102] Peluso, M. J. *et al.* SARS-CoV-2 and mitochondrial proteins in neural-derived exosomes of COVID-19. *Ann. Neurol.* **91**, 772–781. 10.1002/ana.26350 (2022). 35285072 10.1002/ana.26350 PMC9082480

[103] Yonker, L. M. *et al.* Circulating spike protein detected in post-COVID-19 mRNA vaccine myocarditis. *Circulation* **147**, 867–876. 10.1161/circulationaha.122.061025 (2023). 36597886 10.1161/circulationaha.122.061025 PMC10010667

[104] Swank, Z. *et al.* Persistent circulating severe acute respiratory syndrome coronavirus 2 spike is associated with post-acute coronavirus disease 2019 sequelae. *Clin. Infect. Dis.* **76**, e487–e490. 10.1093/cid/ciac722 (2023). 36052466 10.1093/cid/ciac722 PMC10169416

[105] Scheppke, K. A. *et al.* Remission of severe forms of long COVID following monoclonal antibody (MCA) infusions: A report of signal index cases and call for targeted research. *Am. J. Emerg. Med.* **75**, 122–127. 10.1016/j.ajem.2023.09.051 (2024). 37944296 10.1016/j.ajem.2023.09.051

[106] Warth, J. D. *et al.* CFTR chloride channels in human and simian heart. *Cardiovasc. Res.* **31**, 615–624 (1996). 8689654 10.1016/S0008-6363(95)00245-6

[107] Morales, M. M. *et al.* Both the wild type and a functional isoform of CFTR are expressed in kidney. *Am. J. Physiol.* **270**, F1038-1048. 10.1152/ajprenal.1996.270.6.F1038 (1996). 8764323 10.1152/ajprenal.1996.270.6.F1038

[108] Guo, Y., Su, M., McNutt, M. A. & Gu, J. Expression and distribution of cystic fibrosis transmembrane conductance regulator in neurons of the human brain. *J. Histochem. Cytochem.* **57**, 1113–1120. 10.1369/jhc.2009.953455 (2009). 19654104 10.1369/jhc.2009.953455 PMC2778084

[109] Lidington, D. *et al.* CFTR therapeutics normalize cerebral perfusion deficits in mouse models of heart failure and subarachnoid hemorrhage. *JACC Basic Transl. Sci.* **4**, 940–958. 10.1016/j.jacbts.2019.07.004 (2019). 31909302 10.1016/j.jacbts.2019.07.004 PMC6939007

[110] Storstein, L., Nore, A. K. & Sjaastad, O. Studies on digitalis. 23. Blood-brain barrier of digitoxin in humans. *Clin. Cardiol.* **2**, 146–150 (1979). 10.1002/clc.4960020211 262570

[111] Cantin, A. M. *et al.* Cystic fibrosis transmembrane conductance regulator function is suppressed in cigarette smokers. *Am. J. Respir. Crit. Care Med.* **173**, 1139–1144. 10.1164/rccm.200508-1330OC (2006). 16497995 10.1164/rccm.200508-1330OC

[112] Rab, A. *et al.* Cigarette smoke and CFTR: Implications in the pathogenesis of COPD. *Am. J. Physiol. Lung Cell. Mol. Physiol.* **305**, L530-541. 10.1152/ajplung.00039.2013 (2013). 23934925 10.1152/ajplung.00039.2013 PMC3798775

[113] Fernandez Fernandez, E., De Santi, C., De Rose, V. & Greene, C. M. CFTR dysfunction in cystic fibrosis and chronic obstructive pulmonary disease. *Expert Rev. Respir. Med.* **12**, 483–492. 10.1080/17476348.2018.1475235 (2018). 29750581 10.1080/17476348.2018.1475235

[114] Courville, C. A. *et al.* Acquired defects in CFTR-dependent beta-adrenergic sweat secretion in chronic obstructive pulmonary disease. *Respir. Res.* **15**, 25. 10.1186/1465-9921-15-25 (2014). 24568560 10.1186/1465-9921-15-25 PMC4015030

[115] Londino, J. D. *et al.* Influenza virus M2 targets cystic fibrosis transmembrane conductance regulator for lysosomal degradation during viral infection. *FASEB J.* **29**, 2712–2725. 10.1096/fj.14-268755 (2015). 25795456 10.1096/fj.14-268755 PMC4478808

[116] Londino, J. D. *et al.* Influenza virus infection alters ion channel function of airway and alveolar cells: mechanisms and physiological sequelae. *Am. J. Physiol. Lung Cell. Mol. Physiol.* **313**, L845–l858. 10.1152/ajplung.00244.2017 (2017). 10.1152/ajplung.00244.2017 PMC5792181 28775098

[117] Pollard, B. S., Blanco, J. C., Pollard, J. R. & Prince, G. A. Classical drug digitoxin inhibits influenza cytokine storm, with implications for COVID-19 therapy. *bioRxiv*. 10.1101/2020.04.09.034983 (2020). 10.21873/invivo.12221 PMC7811644 33144490

[118] Karki, R. *et al.* Synergism of TNF-α and IFN-γ triggers inflammatory cell death, tissue damage, and mortality in SARS-CoV-2 infection and cytokine shock syndromes. *Cell* **184**, 149-168.e117. 10.1016/j.cell.2020.11.025 (2021). 33278357 10.1016/j.cell.2020.11.025 PMC7674074

[119] Morales, L., Oliveros, J. C., Enjuanes, L. & Sola, I. Contribution of Host miRNA-223–3p to SARS-CoV-induced lung inflammatory pathology. *mBio* **13**, e0313521. 10.1128/mbio.03135-21 (2022). 10.1128/mbio.03135-21 PMC8941895 35229638

[120] Meissner, A. *et al.* Tumor necrosis factor-α-mediated downregulation of the cystic fibrosis transmembrane conductance regulator drives pathological sphingosine-1-phosphate signaling in a mouse model of heart failure. *Circulation* **125**, 2739–2750. 10.1161/circulationaha.111.047316 (2012). 22534621 10.1161/circulationaha.111.047316

