# Supplementary material for: Inflammation in the COVID-19 airway is due to inhibition of CFTR signaling by the SARS-CoV-2 spike protein
Source: Sci Rep. 2024 Jul 23;14:16895. doi: 10.1038/s41598-024-66473-4 (PMC11266487; doi:10.1038/s41598-024-66473-4)
Supplement: Supplementary file 1 — Supplementary Information. [file 41598_2024_66473_MOESM1_ESM.docx]

**Supplemental Information**

**Inflammation in the COVID-19 airway is due to inhibition of CFTR signaling by the SARS-CoV-2 spike protein**

Hung Caohuy ^1,2,3^, Ofer Eidelman (dec) ^1,2^, Tinghua Chen ^1,2,3^, Ognoon Mungunsukh ^1,3,4^, Qingfeng Yang ^1, 5^, Nathan I. Walton ^1,2,3^, Bette S. Pollard ^6^, Sara Khanal ^7,8^, Shannon Hentschel ^7,9^, Catalina Florez ^7,8^, Andrew S. Herbert ^7^, Harvey B. Pollard ^1,2,3,^

1. Department of Anatomy, Physiology and Genetics, Uniformed Services University School of Medicine, Uniformed Services University of the Health Sciences, Bethesda, MD 20814.

2. Collaborative Health Initiative Research Program (CHIRP), Uniformed Services University of the Health Sciences, Bethesda, MD 20814

3. Consortium for Health and Military Performance (CHAMP), Uniformed Services University of the Health Sciences, Bethesda, MD 20814.

4. Center for Military Precision Health, Uniformed Services University of the Health Sciences, Bethesda, MD 20814.

5. Center for the Study of Traumatic Stress (CSTS), and Department of Psychiatry, Uniformed Services University of the Health Sciences, Bethesda, MD 20814

6. Silver Pharmaceuticals, Rockville, MD, 20854

7. United States Army Medical Research Institute of Infectious Diseases (USAMRIID), Virology Division, Fort Detrick, Frederick, MD, 21702

8. The Geneva Foundation, Tacoma WA, 98402

9. Cherokee Nation Assurance, Catoosa, OK 74015

**Supplemental Methods**

Materials and Reagents

All materials and reagents were purchased commercially. β-actin antibody (#A5441), digitoxin, digoxin, ouabain, CFTR_inh_-172, IBMX, amiloride, and L-glutathione (Sigma-Aldrich); CFTR antibodies (University of North Carolina, Chapel Hill, NC UNC450, UNC596, UNC570); ACE2 antibody (Proteintech, #21115-1-AP); alpha-ENaC (#MBS3015153) and gamma-ENaC (#MBS9608857) antibodies (MyBioSource); TRADD (#SC-46653) and IKKβ (#SC-8014) antibodies (SCBT); TNFR1(#3736), IκBα( #9242), phospho-IκBα (Ser32/36) (#9246), phospho-NFκB p65 (Ser276) (#3037), NFκB p65 (#8242), phospho-IKKα/β (Ser176/180) (#2697), and IKKα (#11930) antibodies (Cell Signaling); EZ-Link™ Sulfo-NHS-SS-Biotin (Pierce); polyvinylidene fluoride (PVDF) membrane (Millipore); Human IL-8/CXCL8 DuoSet® ELISA kit (DY208, R&D Systems); Corning Transwell (12-mm, 0.4 mm size pore, 3401) and Snapwell (12-mm, 0.4 mm size pore, 3801) inserts (Fisher Scientific).

With respect to identifying recombinant Spike protein sequences, we have adopted the World Health Organization (WHO) naming system as follows: (i) The spike protein from the original SARS-CoV-2 virus, which expressed aspartic acid at position 614 (*viz*., [D614]) was labeled as Wuhan-Hu-1 [S1S2] spike. (ii) The mutant β-spike 1.351 was labelled as β-1.351 [S1S2] spike. Recombinant Spike proteins were purchased from SinoBiologicals (Philadelphia, PA), and expressed in Baculovirus insect cells with a polyhistidine tag at the C-terminal end. Spike proteins were Wuhan-Hu-1 SARS-CoV-2 [S1S2] spike protein was (Cat # 40589-V08B1); and (ii) β-1.351 [S1S2] protein (K417N, E484K, N501Y, D614G, A701V-His; Cat # 40589-V08811).

Cardiac glycosides were solubilized as 4 mM stock solutions in 100% DMSO, and aliquots were serially diluted in the same solvent, and then into assay medium. In assays containing DMSO, the final solvent concentrations were 0.01% or less.

Cell Cultures and Treatments

Primary human bronchial epithelial (NHBE) cells were purchased from Lonza Bioscience and cultured according to the manufacture’s instructions. CFPAC-1 cells, derived from a pancreatic duct adenocarcinoma from a CF patient homozygous for ΔF508-CFTR, have the ion transport properties of CF-affected epithelia ^1^. Repaired CFPAC-1 4.7 cells, created by transfecting CFPAC-1 cells with the pLJ retrovirus carrying wild-type CFTR ^2^, were kindly provided by Dr. Raymond A. Frizzell. Cells were maintained in high glucose Dulbecco’s modified Eagle’s medium (DMEM) supplemented with10% fetal bovine serum, 2 mM L-glutamine, and antibiotics (100IU/ml penicillin and 100 µg/ml streptomycin) in a 5% CO_2_ environment.

Native SARS-CoV-2 virus infection, cardiac glycoside treatment and western blot.

SARS-CoV-2 β was propagated in Vero E6 cells and used to infect fully differentiated BCi-NS1.1 cells. Prior to infection, titrated virus (200,000 PFU/ 24 mm well) was mixed with ALI growth medium, digitoxin or ouabain (50 nM final concentration) for 1 hour at 37°C. Differentiated cells were infected apically with medium, digitoxin (50 nM), ouabain (50 nM), SARS-CoV-2 (200,000 PFU/ well), SARS-CoV-2 + digitoxin, or SARS-CoV-2 + ouabain. After 4-hour incubation, the mixture media were removed from the apical side, and cells were washed apically twice with PBS and incubated for an additional 20 hours at 37°C and 5% CO_2_ under ALI conditions. Treated cells were washed twice with ice-cold PBS and harvested in RIPA buffer ((1% NP-40, 0.1% SDS, 0.5% deoxycholic acid, 50 mM Tris-HCl (pH 7.4), 150 mM NaCl) supplemented with protease and phosphatase inhibitors. Cell lysates were homogenized using a Bead Mill (VWR, Cat # 10158-558) and spun at 10,000 x g for 20 minutes at 4°C to collect the supernatant. Supernatant protein concentrations were determined by BCA assay (Thermofisher, Cat # 23225). Samples were diluted in 4X NUPage solution (ThermoFisher, Cat # NP0007) and heated at 80 °C for 35 minutes. For each sample, 50 µg of protein were loaded into a 4-12% Bis-Tris pre-cast gel (Thermofisher, Cat # NW04120BOX). Samples were electrophoresed at 200 volts in 1X Mops running buffer (Thermofisher, Cat # NP0001). Gels were transferred onto iBlot 2 NC mini stacks (Thermofisher, Cat # IB23002) with the iBlot 2 gel transfer system (Thermofisher). The membrane was blocked for 2 hours at room temperature while rocking with 5% nonfat milk (Bioworld, Cat # 30620074-1) in 1x PBS and 1% Tween 20 (PBST) (Sigma, Cat # P1379). The membrane was then washed three times for 15 minutes with PBST and was cut into two halves, the top half for probing with a mouse anti-human CFTR C-terminus monoclonal antibody (1:1000, R&D systems, Cat # MAB25031) and the bottom half for probing with a mouse anti-β-actin monoclonal antibody (1:4000, Sigma, Cat # A1978), followed by overnight incubation at 4oC. The membranes were washed as before and incubated with a horseradish peroxidase (HRP)-conjugated goat anti-mouse IgG (1:3000, SeraCara, Cat# 074-1806) for 40 minutes at room temperature. After a final wash, the membranes were incubated with Supersignal substrate (Thermofisher, Cat # 34076,) and imaged using BioRad ChemiDocTM MP touch Imaging System. Protein band densitometry was determined using the Imagelab software (Imagelab 6.1) and CFTR values were normalized to β-actin values.

Ussing chamber analysis

Human hTERT-transformed BCi-NS1.1 basal stem cells were seeded at a concentration of 4.5 x 10^5^ cells/cm^2^ on Snapwell inserts and differentiated at the air-liquid-interface for 25-28 days ^39^. Cell monolayers were exposed to different concentrations of Spike protein for 4 hours, washed with fresh medium, and then incubated for additional 20 hours at 37°C and 5% CO_2_ under ALI conditions. Snapwell inserts were mounted in Ussing Chambers (Physiologic Instruments, Reno Nevada). A basolateral-to-apical chloride gradient was imposed by replacing NaCl with NaGluconate. The chloride-containing solution was 120 mM NaCl, 20 mM NaHCO_3_, 5 mM KHCO_3_, 1.2 mM NaH_2_PO_4_, 1.2 mM CaCl_2_, 1.2 mM MgCl_2_, and 5.6 mM glucose. In the chloride-free solution, all Cl salts were exchanged for gluconate salts, and Ca^+2^ was increased to 5 mM to compensate for the chelation of calcium by gluconate. Both chamber compartments were gassed with 95% O_2_-5% CO_2_ (pH 7.4). Experiments were performed at 37°C. CFTR channel activity was detected by first inactivating sodium currents with 100 μM amiloride; then activating CFTR chloride channels with 10 μM forskolin and 100 μM IBMX; then specifically inactivating CFTR channels with 10 μM CFTR_inh_-172. For Western blotting after Ussing chamber analyses, cells were washed with PBS and lysed in RIPA buffer supplemented with the anti-protease/phosphatase cocktail. Equivalent amounts of lysates (50 μg/sample) were electrophoresed on 4-12% or 4-20% gradient gels (Invitrogen), transferred to PVDF membranes, and membranes were probed with β-Actin antibody (Sigma) or CFTR antibody combo (UNC 450, UNC596, UNC570) at 1:1000 dilution each.

Neutralization Experiment

Differentiated BCi.NS1.1 cells (ALI for 25 days) were treated for 4 hours at 37^0^C with media or 400 ng/ml Wuhan-Hu-1 [S1S2] spike protein in the presence or absence of anti-spike antibody (1:500 dilution or 2 µg/ml; Invitrogen Cat. # PA1-41165) on the apical side. After a 4-hour incubation, cell monolayers were washed with PBS and further incubated at 37^0^C for additional 20 hours under ALI conditions. The inserts were then analyzed for CFTR chloride channel activity by Ussing Chamber analysis, and cells were used for CFTR Western blot analysis after Ussing Chamber measurements.

Enzyme Linked Immunosorbant Assay (ELISA) for interaction between Spike proteins and ACE2

Purified recombinant SARS-CoV-2 Spike proteins were individually dissolved in coating buffer (16 mM Na_2_CO_3_, 34 mM NaHCO_3_, pH 9.6) at a concentration of 2 μg/ml Spike protein. This solution, in 100 μL aliquots, was then added to wells of Costar 96 well plates (Corning, Corning, NY; Catalog # 2592) and incubated overnight at 4^o^C. The next day, wells were washed 3 times in Phosphate Buffered Saline with 0.05% Tween 20 (PBST) at room temperature. Wells were then blocked with 300 μL Blocking Buffer (0.5% Bovine Serum Albumin (BSA), dissolved in PBST) for two hours at 37^o^C. Wells were then washed 3 times in PBST at room temperature (68^o^F). When cardiac glycosides were to be added, they were dissolved in Reagent Diluent (0.5% BSA in PBST), added in 100 μL volumes to each well, and incubated at 37^o^C overnight. Further steps were as described in our recent publication ^29^. The final results are given as averages + SE of all independent experiments.

Statistics

To determine the significance of changes in kinetic parameters of ACE2 binding to SARS-CoV-2 Spike mutants we applied least-squares regression to the linearized Eadie-Hoffstee plot of the binding data, and determined the statistical significance of the difference between the slopes (*i.e*., K_D_’s) and between the intercepts (*i.e*., B_max_) using R or Stata statistical packages. K_i_ values were calculated for each data point in the linear range depending on the inhibition mechanism. Except as noted, all plotted data points are the means of 3 or more independent experiments. Means ± SE were calculated; *p* < 0.05 were taken to indicate a significant difference from controls. For analyses of Ussing chamber and Western blot experiments the data are expressed as means ± SD. Statistical *p* values were determined with a one-way ANOVA, followed by Holm’s post-hoc test for multiple comparisons, by comparing each mean to the medium control and compared for consistency with Dunnett’s post-hoc tests as noted in Figure legends. In each case significance was assigned if *p* < 0.05.

Supplemental References

1 Schoumacher, R. A. et al. A cystic fibrosis pancreatic adenocarcinoma cell line. Proc. Natl. Acad. Sci. U.S.A. 87, 4012-4016 (1990). doi: 10.1073/pnas.87.10.4012.

2 Drumm, M. L. et al. Correction of the cystic fibrosis defect in vitro by retrovirus-mediated gene transfer. Cell 62,1227–1233 (1990). doi: 10.1016/0092-8674(90)90398-x.

**Supplemental Figures**

**Supplemental Figure S1**

**Figure S1**. **Inactivation of CFTR by spike protein activates both NFκB p65 and α,β,γ ENaC signaling**. Under baseline conditions CFTR tonically suppresses ENaC and TRADD. However, upon addition of [S1S2] spike, CFTR is dose-dependently reduced by failure to be recovered from endosomal recycling (this paper). In the absence of CFTR, TRADD is no longer constitutively directed to the proteosome, and it is free to activate IKKαβγ, which phosphorylates IκBα. NFκB p65 is now free to leave the cytosol and enter the nucleus. Cytokines and chemokines such as IL-6, IL-8 and mTNFα are then expressed. Membrane-bound TNFα (mTNFα) is converted to soluble sTNFα/TNFα by ADAM17/TACE). ADAM17/TACE also converts membrane bound mACE2 to soluble sACE2. Cardiac glycoside drugs ouabain, digitoxin and digoxin are potent competitive inhibitors of spike:ACE2 binding (Caohuy H. et al, Scientific Reports,2021). Digitoxin separately blocks interactions in the host between the TNFα/TNFR1 complex and TRADD (Yang Q et al, PNAS, 2005). In the absence of CFTR, ENaC is also proteolytically activated by FURIN and TMPRSS2 (this paper). In preparation for penetration into the target cell, the same proteases cleave Spike at the S1S2 junction. Color code: **red** = elevated; **green** = reduced; black = no known change.

**Supplemental Figure S2**

**Supplemental Figure S2. Kinetic of CFTR channel activity and CFTR protein expression affected by Wuhan-Hu-1 and β-1.351 [S1S2] spike protein variants. (a)** CFTR Channel conductance as a function of Wuhan-Hu-1 or β-1.351 [S1S2] spike concentrations. K_i_ forWuhan-Hu-1 spike is 448 ng/ml (R^2^ = 0.9898). K_i_ for β spike is 210 ng/ml (R^2^ =0.9956). (**b).** CFTR protein level as a function of Wuhan-Hu-1 and β spike concentrations. K_i_ for Wuhan-Hu-1 spike is 399 ng/ml (R^2^ =0.9842). K_i_ for β spike is 193 ng/ml (R^2^ = 0.9590). Graphs are derived from the Eadie-Hoffstee equation. K_i_ values are the slopes of the straight lines. Points are averages from N=4 ± SE independent experiments.

**Supplemental Figure S3**


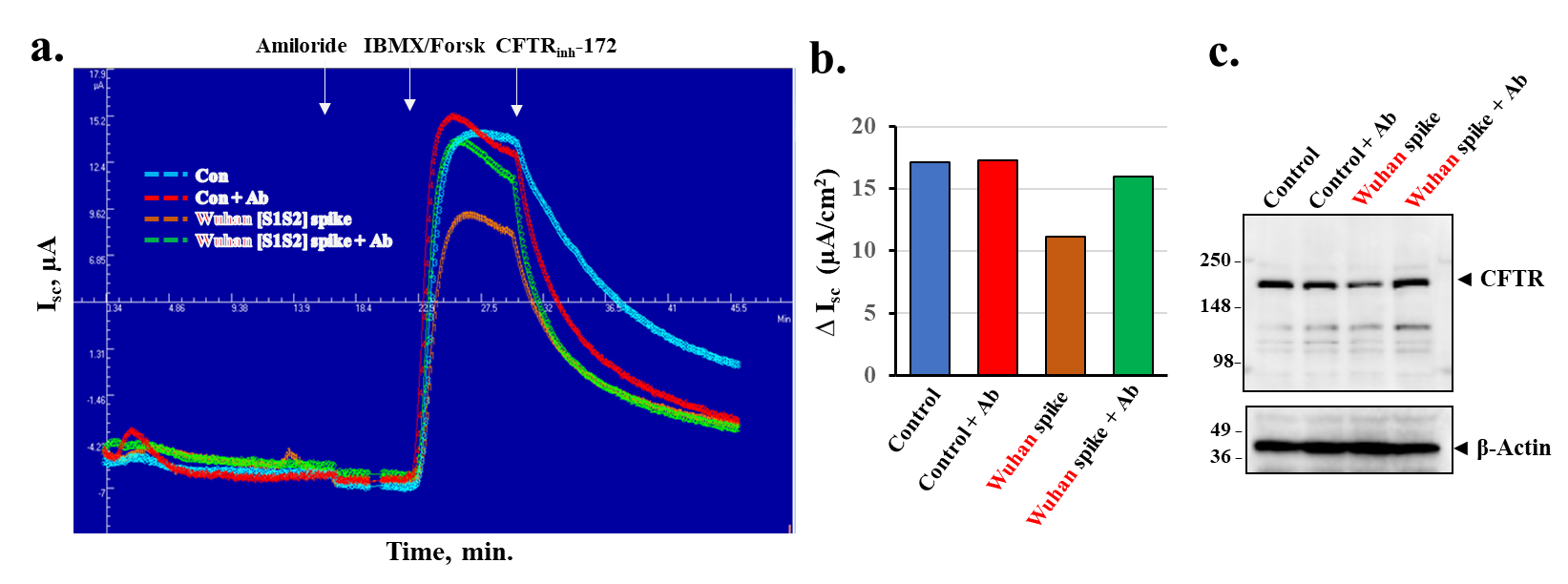


**Supplemental Figure S3. Anti-**s**pike antibody (Ab) neutralizes the inhibitory action of Wuhan-Hu-1 [S1S2] spike protein on cAMP-activated CFTR chloride channel activity in differentiated NCi.S1.1 cells.** Differentiated BCi.NS1.1 cells (ALI for 25 days) were treated for 4 hours at 37^0^C with media or 400 ng/ml Wuhan-Hu-1 [S1S2] spike in the presence or absence of anti-spike antibody (2 μg/ml) on the apical side, washed, and further incubated at 37^0^C for additional 20 hours under ALI conditions. CFTR-dependent short-circuit currents (I_SC_) were measured in Ussing Chambers as the changes in reponse to Amiloride, IBMX/Forskolin, and CFTR_inh_-172. (**a**) Representative current I_SC_ tracings, (**b**) a summary of changes in I_sc_ of two independent Ussing chamber experiments, and (**c**) A representative Western blot image of CFTR expression in treated cells after Ussing Chamber analyses of two independent experiments are shown. β-Actin was used for equal loading of protein.

**Supplemental Figure S4**


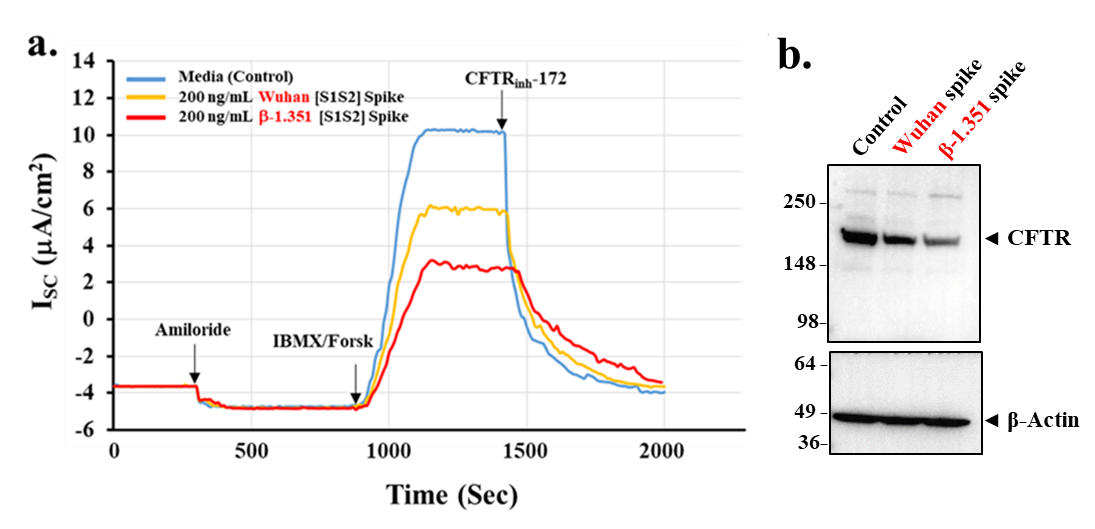


**Supplemental Figure S4. Wuhan-Hu-1 and β-1.351 [S1S2] spike proteins reduce cAMP-activated CFTR chloride channel activity in differentiated primary human bronchial epithelial (NHBE) cells.** Primary NHBE cells purchased from Lonza were cultured according to the manufacture instructions and and differentiated on Snapwell inserts under the ALI conditions for 28 days. Differentiated primary HBE cells were exposured to 400 ng/mL Wuhan-Hu-1 or β-1.351 [S1S2] spike protein on the apical side for 4 hours, washed and then incubated for additional 20 hours under ALI conditions. CFTR-dependent short-circuit currents (I_sc_) were measured in Ussing Chambers as the changes in response to Amiloride, IBMX/Forskolin, and CFTR_inh_-172. (**a**) Representative current I_sc_ tracings, and (**b**) A representative Western blot image of CFTR expression in treated cells after Ussing Chamber analyses of two independent experiments are shown. β-Actin was used for equal loading of protein.

**Supplemental Figure S5**

**Supplemental Figure S5. SARS-CoV-2 spike-induced changes in CFTR protein expression in CFPAC 4.7 cells and rescue by cardiac glycoside drugs.** CFPAC 4.7 cells were treated with 400ng/ml SARS-COV2 [S1S2] spike protein, or with 50nm Digitoxin (Digit), Digoxin (Dig) and Ouabain (Ouab), respectively for 4 hours in Serum-free DMEM medium. Following a wash with serum-free DMEM medium, cells were incubated in the full DMEM medium (with 10% FBS) for 20 hours. Cells were lysed with 1x RIPA buffer with protease inhibitors. 15ug of each sample was loaded for Western blotting analysis. (**a&c).** SARS-CoV-2 [S1S2] spike protein-induced CFTR protein expression in CFPAC4.7 cells. Both Wuhan-Hu-1 and β-1.351 caused significant reductions in CFTR expression. (**b&d).** Cardiac glycoside drugs prevented CFTR reduction caused by SARS-CoV-2, strain β-1.351 [S1S2] spike protein on CFPAC4.7 cells. Data represent averages of three independent experiments. Statistically significant differences from control are based on * (p < 0.05), *** (p < 0.001).

**Supplemental Figure S6**

**
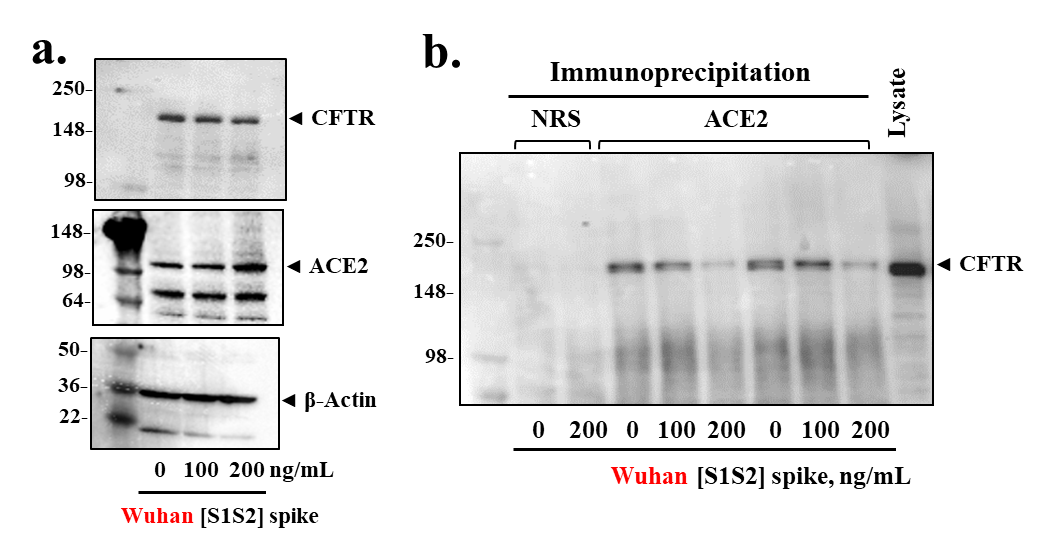
**

**Supplemental Figure S6. Influence of Spike protein on ACE2 and CFTR co-immunoprecipition in differentiated epithelia. (a)** Increasing concentrations of Wuhan-Hu-1 [S1S2] spike protein cause loss of CFTR but an increase in ACE2. (**b**) Anti-ACE2 antibody co-immunoprecipitates CFTR from differentiated (d-BCi) epithelia lysates. Pre-treatment of cultures with increasing concentrations of Wuhan-Hu-1 [S1S2] spike protein results in loss of CFTR. NRS is control normal rabbit serum. Western blot data represent the results of three independent experiments.

**Supplemental Figure S7**

**Supplemental Figure 7. CFTR Endosomal Recycling Process.** CFTR proteins on the epithelial cell surface are internalized by a Clathrin/ Dynamin-dependent process that depends on the Ras-related protein RAB5 for movement of CFTR to early endosomes. Myosin 6 drives physical internalization by binding to the (+) end of actin and moving cargo towards the (-) actin end. RAB5 recruits RAB7 to drive maturation of early endosomes to late endosomes. Maturation includes transporting vacuolar (H+) ATPases (V-ATPases) from the Trans-Golgi Network (TGN) to the endocytic vesicles. RAB4 mediates quick return of CFTR-laden endosomes to the plasma membrane. RAB11 mediates slower return to the plasma membrane. Late endosomes are also conveyed to the lysosome for destruction of “damaged” CFTR. SARS-CoV-2 Spike protein drives destruction of cell surface CFTR by this endosomal recycling process. RAB9 mediates transfer of CFTR from Late endosome to the Trans-Golgi-Network (TGN). Grey squares represents CFTR. Fragmented grey squares represents proteolysis of CFTR in the lysosome. (Adapted from Ameen, Silvis and Bradbury, 2007).

**Supplemental Figure S8 (Text Fig1 + original Westerns )**

**Supplemental Figure S9 (Text Fig3 + original Westerns )**

**Supplemental Figure S10 (Text Fig4 + original Westerns )**

Beta-Actin

**Supplemental Figure S11 (Text Fig5 + original Westerns )**

**Supplemental Figure S12 (Text Fig6 + original Westerns )**

**Supplemental Figure S13 (Text Fig7 + original Westerns )**

**Supplemental Figure S14 (Text Fig8 + original Westerns )**


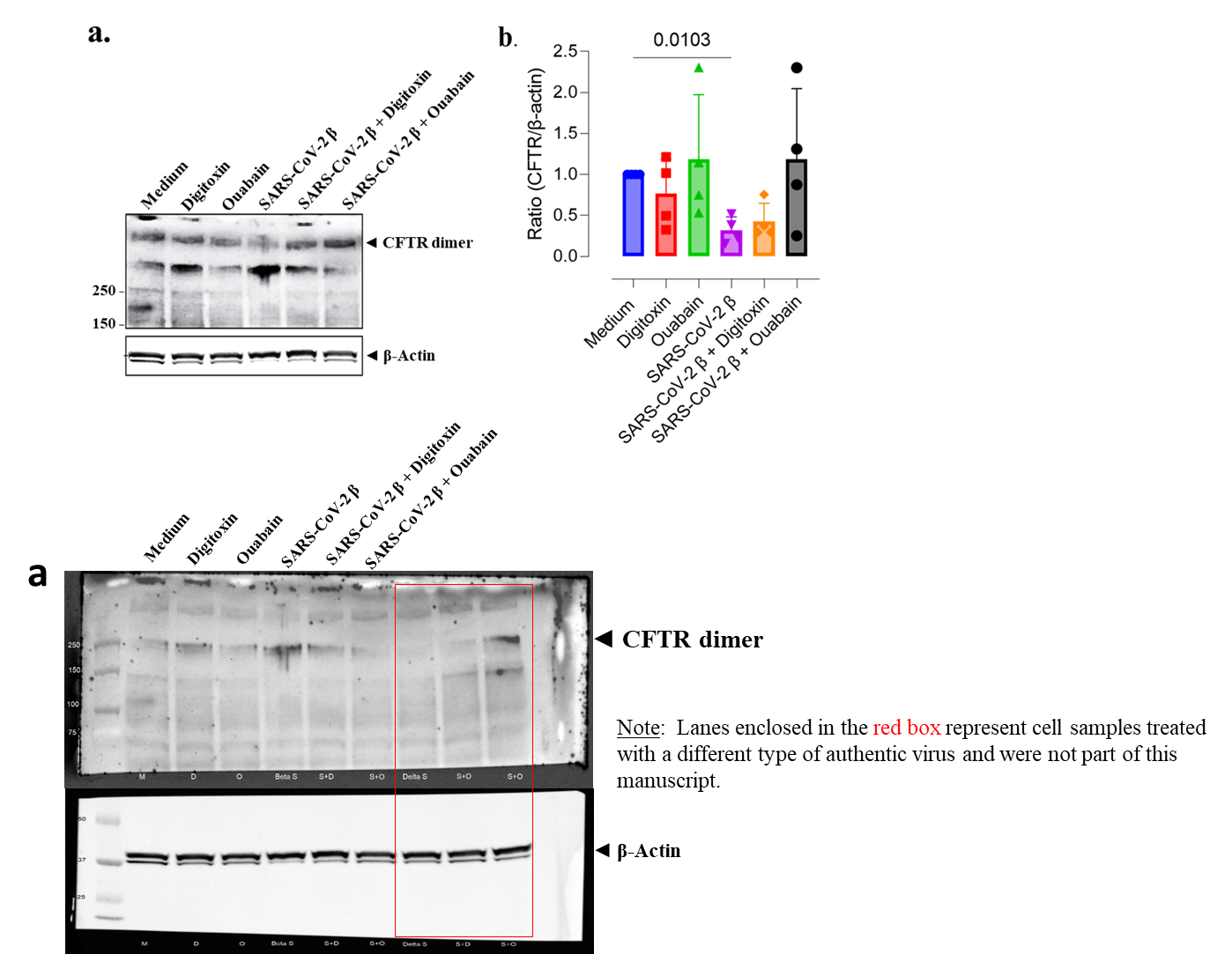


**Supplemental Figure S15 (Supplemental FigS3 + original Westerns )**

**Supplemental Figure S16 (Supplemental FigS4 + original Westerns )**

**Supplemental Figure S17 (Supplemental FigS5 + original Westerns )**

**Supplemental Figure S18 (Supplemental FigS6 + original Westerns )**
